# Physiologically Based Pharmacokinetic Modelling and Simulation to Predict the Plasma Concentration Profile of Doxorubicin

**DOI:** 10.3390/pharmaceutics14030541

**Published:** 2022-02-28

**Authors:** George A. Mystridis, Georgios C. Batzias, Ioannis S. Vizirianakis

**Affiliations:** 1 Laboratory of Pharmacology, School of Pharmacy, Faculty of Health Sciences, Aristotle University of Thessaloniki, GR-54124 Thessaloniki, Greece; geormyst@pharm.auth.gr; 2 Laboratory of Pharmacology, School of Veterinary Medicine, Faculty of Health Sciences, Aristotle University of Thessaloniki, GR-54124 Thessaloniki, Greece; batzias@vet.auth.gr; 3 Department of Life and Health Sciences, School of Sciences and Engineering, University of Nicosia, Nicosia CY-1700, Cyprus

**Keywords:** doxorubicin, physiologically based pharmacokinetic model, pharmacokinetics, simcyp simulator, PBPK modelling

## Abstract

Doxorubicin (DOX) is still an important anticancer agent despite its tricky pharmacokinetics (PK) and toxicity potential. The advent of systems pharmacology enables the construction of PK models able to predict the concentration profiles of drugs and shed light on the underlying mechanisms involved in PK and pharmacodynamics (PD). By utilizing existing published data and by analysing two clinical case studies we attempt to create physiologically based pharmacokinetic (PBPK) models for DOX using widely accepted methodologies. Based on two different approaches on three different key points we derived eight plausible models. The validation of the models provides evidence that is all performing as designed and opens the way for further exploitation by integrating metabolites and pharmacogenomic information.

## 1. Introduction

Despite being an “old” drug, Doxorubicin (DOX) still remains an important and valuable therapeutic agent in cancer therapy [1]. Its clinical use, however, is limited due to safety issues. The latter is correlated to the cumulative dose used and is manifested mainly as cardiotoxicity that could potentially lead to congestive heart failure (CHF) [2] causing up to 50% mortality [3]. There are two proposed mechanisms that are correlated to the plasma levels of its two metabolites doxorubicinol (DOXol) and doxorubicinone (DOXone), although there is still a controversy, due to the pleiotropic pharmacological effects of DOX at the molecular level and complex pharmacokinetic (PK) profile [4,5,6]. Both metabolites produce reactive oxygen species (ROSs) intracellularly that trigger cytotoxicity and programmed cell death [4,5,6]. Of special research interest is also the fact that the clinical outcome shown by DOX exhibits a sex-related behaviour, an observation that remains elusive and challenges the scientific community [7].

The pegylated liposomal DOX formulation (Doxil^®^, Caelyx^®^) that entered the market in 1995 represents the first FDA-approved nanomedicine that successfully addressed the problematic PK behaviour and specific safety profiles of DOX [8,9]. Specifically, it showed enhanced circulation time of DOX as well as a better tumour accumulation profile [10,11]. Recently, quantitative systems pharmacology (QSP) is in the spotlight of modern pharmacology by providing, through physiology-based pharmacokinetic (PBPK) modelling, mechanistic insights on the PK processes towards achieving better efficacy and safety profiles in the clinical setting [12]. PBPK models are based on in vitro-in vivo correlation (IVIVC) procedures. Striving to be as mechanistic as possible in nature, they are based on the underlying anatomical, physiological, and biochemical characteristics of an organism [13]. In such models, the body is a multicompartment system with every compartment representing a different organ connected to other compartments by blood or lymph circulation, through a system of differential equations describing different phenomena, such as blood flow, cardiac output, organ volumes, glomerular filtration rate etc. [14].

Such capability gives the advantage to PBPK models and leads to better identification of the sources of PK variability allowing to extrapolate to different subpopulations [10]. In this context, precision medicine could be achieved in the clinical setting by connecting PBPK models with pharmacodynamic (PD) prediction models and their capacity for population simulation [i.e., the prediction of the effects of age, gender, comorbidities, genetic polymorphisms, lifestyle factors (e.g., smoking) and more] [13].

Finally, an advantage of such models is their ability to simulate the drug concentration profile on the site of action (e.g., targeted organ or tissue), allowing the refinement of dosage schemes and the achievement of maximum safety and effectiveness profiles [15]. This can be of great value in the case of advanced formulations, such as nanoformulations, as the combination of knowledge about the structure and the function of target organs, with the physicochemical properties of the nanocarriers, the individual parameters of each patient and the drug properties could create favourable conditions for individualized treatment [16].

## 2. Materials and Methods

To address the issues related to therapeutic peculiarities related to DOX and its metabolites in the body we attempt the development of a PBPK model capable of predicting the PK profile of DOX in the plasma. This first step presented in our work, provides a solid basis for further incorporation of DOX main metabolites [Doxorubicinol (DOXol) and Doxorubicinone (DOXone)] kinetics in the future. With a working model at hand, adjustments will be possible so that the potential application of such a PBPK model in various innovative DOX nano-formulations, e.g., Doxil^®^, could be a useful clinical tool in treating cancer patients. The ability of the clinicians to estimate, through such a PBPK model, the proper dose of DOX to achieve the maximum efficacy and safety profiles in individual patients is fundamental within the precision medicine concept. 

**Figure 1:**
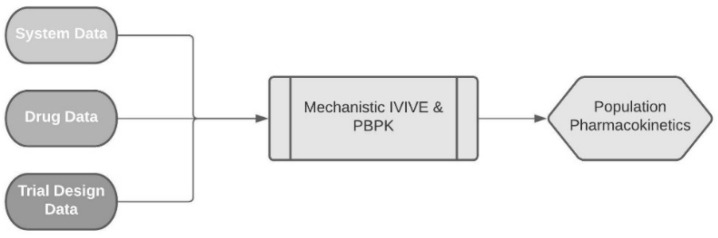
Depiction of the three independent but interacting parts of a PBPK model. Adapted from Jamai et al. [21] which is licensed under a Creative Commons Attributions (CC BY 4.0) International License (http://creativecommons.org/licenses/by/4.0/).

There are several attempts in the literature trying to develop PBPK models for DOX; however, with different end goals. Dubbelboer et al. created both PBPK and semi-PBPK models for DOX attempting to incorporate intracellular binding as a distribution factor in the latter one [17]. Gustafson et al. created a mouse PBPK DOX model with macromolecule-specific binding as the main factor for distribution and organ-specific metabolism and excretion. These authors believe their model has the potential to predict the magnitude of PK interactions of DOX with other drugs, as well as more efficiently addressing various clinical situations [18]. Hanke et al. developed a PBPK model for a DOX fusion molecule, namely “zoptarelin doxorubicin”. To this end, they initially developed a DOX PBPK model (as DOX is the metabolite of the fusion molecule). They also utilized DNA intracellular binding to predict distribution. They had unspecified hepatic clearance and bile elimination [19]. He et al. created a PBPK model to assess DOX disposition at many levels (system, tissue interstitial, cell and subcellular organelles) by analyzing mice data and scaling up for humans thus gaining insights concerning toxicity [20]. In our approach, various relevant models were created and validated using a middle-out approach. In this approach, existing clinical observations are utilized in a reverse translation approach to combine any prior information on the drug or system into the analysis of the clinical observations to project forward beyond the scope of the initial observations. In such methodologies, the models comprise of three different interacting components, the system data, the drug data, and the clinical trial data. System data refers to the properties of the organism for which the model is created (e.g., blood flow or enzyme activity). Drug data refers to the physicochemical data and PK information of the modelled drug (e.g., drug pK_a_, affinity for enzymes, etc.). Clinical trial data refers to the administration settings (e.g., population age, female percentage, dosage scheme, etc.) [21]. The interaction of these three components is summarized in Figure 1.

### 2.1. Clinical Studies Used

To construct a model two types of datasets are needed: (a) a training dataset, utilized in the development of the model and (b) a validation dataset, used for independent validation of the model. For our models, we used the clinical study of Camaggi et al. as the training dataset [22] and the clinical study of Speth et al. as the independent validation dataset [23]. The details of those clinical studies are summarized in Table 1.

### 2.2. Software

The Simcyp (version 19R1) simulator (Simcyp Ltd., Sheffield, UK) was used to simulate the PK profile of DOX. The criteria for assessing the predictive performance of the models were the predicted/observed ratio for the AUC and the C_max_ of DOX. A perspective on the qualification and verification of PBPK models, as well as examples of regulatory PBPK submissions, can be found in the work of Shebley et al. [24].

### 2.3. Virtual Population Characteristics (System Data)

As both clinical studies utilized in this work (training and validation dataset) refer to a cancer population, we opted for the cancer population (Sim-Cancer) that is included within the Simcyp simulator. For this special population, many adjustments have been made to better account for the specific changes that are expected to be found in the physiological parameters of such a population.

### 2.4. Development of DOX PBPK Model (Drug Data)

To construct the models, we need to input the physicochemical properties of DOX, values for DOX renal, metabolic, and biliary clearance and to select an appropriate model for DOX distribution. The physicochemical properties of DOX were either calculated, found by literature review or online database utilization. The pharmacokinetic parameters were calculated based on the data from the training dataset. For the calculations, two patients of the original case study were excluded from further analysis: (a) Patient 69 because of hepatic metastases, extrahepatic obstruction, and percutaneous biliary drainage and (b) Patient 72 as the hepatic clearance exceeded the hepatic blood flow that was calculated. The calculated pharmacokinetic parameters are included in Supplementary Materials Section S1. The values are summarized in Table 2.

#### 2.4.1. Calculating DOX Renal Clearance

The Simcyp simulator requires renal clearance to be inputted as the renal clearance of a healthy 20–30 y.o. male (*refCL_R_*). We calculated the *refCL_R_* based on the patient’s GFR and the GFR values expected for 20–30 y.o. males as shown in Equation (1).

(1) $$refCL_{R,i}=\frac{CL_{R,i}\times GFR_{ref}\;}{eGFR_{i}}$$

where *refCL_R,i_* is the reference renal clearance for each patient *i*, *CL_R,i_* is the renal clearance of each patient *i*, *GFR_ref_* is the reference GFR for a 20–30 y.o. male and *eGFR_i_* is the expected GFR for each patient *i*.

As GFR values per patient were not mentioned in the work of Camaggi et al. used as a training dataset [22], we calculated those values based on two methods: Method A and Method B.

##### Method A

In this method, we used the approach of Davies and Shock [25] for calculating the expected GFR (*eGFR*) for each patient *i* by utilizing expected GFR values per age group (Equation (2)).

(2) $$eGFR_{i}=\frac{GFR_{g,i}\times BSA_{i}}{1.73}\frac{\mathrm{mL}}{\min}$$

where *eGFR_i_* is the expected GFR for each patient *i*, *GFR_g,i_* is the expected GFR for the group that each patient *i* belonged based on age, and *BSA_i_* is the is body surface area of each patient *i*, a value that was recorded in the training dataset clinical study.

##### Method B

In this method, we used the approach of Wright et al. [26] and calculated the expected GFR values based on the expected serum creatinine levels of each patient *i* (Equation (3)).

(3) $$eGFR_{i}=\frac{\left(6580-38.8\times age_{i}\right)\times BSA_{i}\times \left(1-0.168\times SEX_{i}\right)}{SCr_{i}}$$

where *eGFR_i_* is the expected GFR, *age_i_* is the age and *SCr_i_* is the serum creatinine levels of each patient *i*. The parameter *SEX_i_* takes two distinct values, 0 for males and 1 for females based on the gender of each patient *i*.

However, as the gender of each patient *i* was not included in the study, an average GFR value was calculated by averaging the GFR values assuming both male and female gender (Equation (4)).

(4) $$eGFR_{i,avg}=\frac{eGFR_{i,m}+eGFR_{i,f}}{2}$$

where *eGFR_i,m_* is the estimated GFR values for patient *i* if considered male and *eGFR_i,f_* if considered female.

The calculated values of renal clearance of DOX for a 20–30 y.o. healthy male (*refCL_R_*) are summarized in Table 2. For the values of each individual patient for each method, see Supplementary Materials Section S3. It is noteworthy that the calculated renal clearance for DOX exceeds the calculated GFR values, thus implicating the involvement of active processes in renal excretion, a fact that is discussed below in the model limitations.

#### 2.4.2. Calculating DOX Hepatic Clearance

The Simcyp simulator can calculate the hepatic clearance of a drug by scaling using different in vitro systems based on the following method:

$$CLu_{int}\;\left(in\;vitro\;system\right)\overset{Scaling\;Factor\;1}{\to }\;CLu_{int}\;per\;gram\;liver\overset{Scaling\;Factor\;2}{\to }\;CLu_{int}\;per\;liver\;$$

Hepatic metabolic clearance can be predicted using either hepatocyte, cytosolic fraction, or microsomal fraction in vitro systems. Biliary excretion can be calculated using the hepatocytes in vitro system. Thus, to be able to predict the hepatic clearance for a simulated patient, we must calculate the hepatic intrinsic clearance first and then we must calculate the intrinsic metabolic clearance and the intrinsic biliary excretion and correct them by the appropriate scaling factors as seen in Table 3.

To calculate the hepatic intrinsic clearance, we can use Equation (5) which is based on the well-stirred liver model.

(5) $$CL_{H,b,i}=Q_{H,i}\times E_{H}=Q_{H,i}\times \frac{fu_{b}\times CLu_{\;int,H,b,i}}{Q_{H,i}+fu_{b}\times CLu_{\;int,H,b,i}}$$

where *CL_H,b,i_* is the hepatic blood clearance for each patient *i*, *Q_H,i_* is the hepatic blood flow of each patient *i*, *fu_b_* is the unbound fraction of the drug in blood and *CLu_int,H,b,i_* is the intrinsic hepatic clearance of the unbound drug for each patient *i*. 

By rearranging Equation (5), we obtain Equation (6) and thus the intrinsic clearance of the unbound drug can be calculated.

(6) $$CLu_{int,H,b,i}=\frac{Q_{H,i}\times CL_{H,b,i}}{fu_{b}\times \left(Q_{H,i}-CL_{H,b,i}\right)}$$

From the above, it is obvious that there are three parameters that need to be calculated in order to calculate the hepatic intrinsic unbound clearance of a drug, namely the (1) hepatic blood flow of each patient *i*, *Q_H,i,_*, (2) the unbound fraction of a drug in blood, *f_u,b_* and (3) the hepatic blood clearance for each patient *i*, *CL_H,b,i_*.

##### Calculating the Hepatic Blood Flow for Each Patient *i*

Blood is supplied to the liver via two paths: (a) through the hepatic artery and (b) through the portal vein. In the virtual population we selected for our models (i.e., cancer population), the liver receives a predefined percentage of the cardiac output ($f_{CO_{L}})\;$via the above-mentioned methods, different for each gender. As the clinical study used as the training dataset did not include gender, the average percentage for the two genders was used, as shown in Table 4.

In the cancer population used for the models, the formula for calculating the cardiac output for each patient *i* is a function of body surface area (BSA) and age as shown in Equation (7).

(7) $$CO_{i}=BSA_{i}\times 60\times \left(3-\frac{age_{i}-20}{100}\right)$$

where *CO_i_* is the cardiac output of each patient *i*, *BSA_i_* is the body surface area of each patient *i* and *age_i_* is the age of each patient *i*. 

Thus, the hepatic blood flow for each patient *i* (*Q_H,i_*) can be calculated using Equation (8).

(8) $$Q_{H,i}=CO_{i}\times f_{CO_{L}}=CO_{i}\times 0.2675$$

For the values of the cardiac output calculated for each individual patient, see Supplementary Materials Section S4.$\;f_{CO_{L}}\;$represents the percentage of *CO* for the liver and is calculated in Table 4.

##### Calculating Unbound Fraction in Blood

We know that for the unbound fraction of a drug in blood, Equation (9) applies.

(9) $$f_{u}\times C_{p}=f_{u,b}\times C_{b}\;↔\;f_{u,b}=f_{u}\times \frac{C_{p}}{C_{b}}=f_{u}\times R_{p:b}\;$$

As we mentioned earlier, the unbound fraction of DOX in blood was 0.25 and the B:P ratio (*R_B:P_*) was calculated to be 0.87 (see Supplementary Materials Section S5). Thus, for DOX, *f_u,b_* was calculated to be 0.2175 as shown here: $f_{u,b}=0.25\times 0.87=0.2175\;$.

##### Calculating Hepatic Blood Clearance

Hepatic blood clearance (*CL_H,b_*) can be calculated for each patient *i* by utilizing Equation (10):

(10) $$CL_{H,b,i}=\frac{CL_{H,i}}{R_{B:P}}$$

where *CL_H,i_* is the hepatic plasma clearance for each patient *i* and *R_B:P_* is the ratio of DOX concentration in blood vs. plasma. Thus *CL_H,B_* was calculated for each patient *i*. For the values of each individual patient, see Supplementary Materials Section S6.

##### Calculating Intrinsic Hepatic Clearance

Using the values for the hepatic blood flow for each patient *i*, the unbound fraction of DOX in blood and the values of hepatic blood clearance calculated for each patient *i* in Equation (6), we calculated *CLu_int,H,b_* for each patient *i*.

##### Separating Hepatic Clearance to Hepatic Metabolic Clearance and Biliary Excretion

Based on the literature [27], approximately 40% of DOX is excreted in the bile as unchanged drug while 5–12% of DOX and its metabolites are excreted in urine. In the training datasets, the patient had an average plasma clearance (*CL_P_*) of 51.75 L/h, an average renal clearance (*CL_R_*) of 5.59 L/h, and thus an average fraction excreted in urine (*f_e_*) of 11.07%. (For detailed calculations see Supplementary Materials Section S7). Based on the above, the fractions that are eliminated via different routes used for the construction of the model are presented in Table 5.

Thus, the biliary excretion can be calculated to be 44.94% of the hepatic clearance ($f_{CL,H,bile})\;$by using Equation (11) and the values from Table 5.

(11) $$f_{CL,H,bile}=\frac{f_{bile}}{1-f_{e}}$$

where *f_bile_* is the percentage of DOX excreted in bile and *f_e_* is the percentage of DOX excreted in urine as unchanged drug.

By assuming that the ratio of biliary clearance to hepatic clearance is the same as intrinsic biliary clearance to intrinsic hepatic clearance, we can calculate the intrinsic biliary clearance and intrinsic metabolic clearance by the intrinsic hepatic clearance we calculated before for each patient *i*. For the values of each patient, see Supplementary Materials Section S8.

##### Correction of Intrinsic Biliary Clearance for In Vitro System Scaling Factors

Intrinsic biliary clearance must be corrected for Scaling Factor 2 (i.e., liver weight—LW) and Scaling Factor 1 (in this case, hepatocellularity per gram liver—HPGL). The correction is carried out using Equation (12).

(12) $$CLu_{int\left(Βile\right),i}=\frac{CLu_{int,bil,b,i}}{LW\times HPGL_{i}\;}$$

where *CLu_int(Bile),i_* the biliary excretion per million hepatocytes for each patient *i*, *CLu_int,bil,b,i_* the intrinsic biliary excretion for each patient *i* and *HPGL_i_* the hepatocellularity per gram liver for each patient *i*. The individual values calculated and information about calculating HPGL for each patient can be found in Supplementary Materials Section S9. The average value of unbound intrinsic metabolic clearance per million hepatocytes is summarized in Table 2.

##### Calculating Intrinsic Metabolic Clearance

As was the case with intrinsic biliary excretion, again in the case of intrinsic metabolic clearance, we must correct the values for both Scaling Factor 2 (i.e., LW) and Scaling Factor 1 (i.e., HPGL, MPPGL or CPPGL). Here, one can follow two approaches: either assign all metabolic clearance to be predicted using HPGL (Method C) or by considering the metabolic pathway of DOX, try to distribute it to different in vitro systems based on the location of the actual metabolizing enzymes (Method D). The latter will be useful as it can be utilised to simulate the formation of the different DOX metabolites in the future.

The following equations are used for the calculation of each in vitro system:

(13) $$CLu_{int\left(Μet\right),HEP,i}=\frac{CLu_{int,met,b,i}}{LW\times HPGL_{i}}$$

(14) $$CLu_{int\left(Μet\right),HLM,i}=\frac{CLu_{int,met,b,i}}{LW\times MPPGL_{i}}$$

(15) $$CLu_{int\left(Met\right),HLC,i}=\frac{CLu_{int,met,b,i}}{LW\times CPPGL_{i}}$$

The values for correcting the entire intrinsic metabolic clearance for the three in vitro systems are summarized in Table 2. Values for MPPGL and HPGL come from the literature [28,29] while the Simcyp simulator calculates values for CPPGL. Details of calculating individual HPGL, MPPGL and CPPGL values as well as the values for each patient are in Supplementary Materials Section S9. Individual patient values for biliary excretion are in Supplementary Materials Section S10. The corrected values for intrinsic metabolic clearance for each patient are in Supplementary Materials Section S11.

Table 6 shows the final values of intrinsic metabolic clearance for each in vitro system when considering the expected relative contribution of each based on the DOX metabolic pathway. This distribution was performed by considering the following facts [27]:

- The enzymes that participate in the primary metabolic pathway, which is the two-electron reduction, mainly aldoketoreductases (AKRs) and carbonylreductases (CBRs) are located in the cytoplasm and are expected to be found in the cytoplasmic fraction after centrifugation.
- The enzymes that participate in the secondary metabolic pathway, which is the one-electron reduction, are located mainly in mitochondria and sarcoplasmic reticulum and are expected to be found in the microsomal fraction after centrifugation.
- The enzymes that participate in the minor metabolic pathway, which is the deglycosylation, are not specified, contribute only 1–2% of the total metabolism and thus their contribution can be attributed per 10^6^ hepatocytes.

#### 2.4.3. Selecting a Distribution Model for DOX

To simulate DOX distribution, either the minimal PBPK (mPBPK) model or the full PBPK (fPBPK) models can be used. Both are models provided by the Simcyp simulator to predict the distribution of drugs. In the case of the mPBPK, the V_ss_ value for DOX was user-inputted based on the values of the training dataset. In the case of the fPBPK model, V_ss_ was calculated in silico. The V_ss_ values are summarized in Table 2. Thus, there are two methods for calculating DOX distribution, either using the mPBPK model (Method E) or the fPBPK model (Method F).

By using the Simcyp parameter estimation (PE) tool, the appropriate parameters were calculated (i.e., the V_sac_ volume (L) and V_sac_ blood flow (L/h) in the case of mPBPK and the K_p_ scalar in the case of fPBPK). The calculated values are summarized in Table 7. For more information on distribution models utilized by Simcyp, see Supplementary Materials Section S12.

#### 2.4.4. Generating DOX Models

Summarizing all the above, we have two different approaches on three key calculations thus resulting in eight possible DOX models. The models are presented in Table 7.

**Table 7:** Different possible DOX models based on two different approaches on three key points.

Model	*CL_R_* (L/h)	*CL_met_*	Distribution Model	P.E. K_p_ Scalar	P.E. V_sac_ (L/Kg)	P.E. Q_sac_ (L/h)
1	7.04	HEP	mPBPK	NA	31.5495	151.3618
2	7.04	HEP	fPBPK	5.3119	NA	NA
3	7.04	DIST	mPBPK	NA	31.3833	212.3364
4	7.04	DIST	fPBPK	5.3119	NA	NA
5	8.67	HEP	mPBPK	NA	31.2603	237.3875
6	8.67	HEP	fPBPK	5.237	NA	NA
7	8.67	DIST	mPBPK	NA	31.3827	211.6487
8	8.67	DIST	fPBPK	5.3119	NA	NA

NA: not applicable; *CL_met_*: represents the metabolic clearance for each model calculated either by 10^6^ hepatocytes (HEP) or using our custom distribution (DIST) of metabolic clearance on in vitro systems (see Table 6).

### 2.5. Virtual Patient Demographics for the Development of the Model Based on the Training Dataset (Clinical Settings Data)

The age of the virtual patients was set from 42 to 72 years to match the age of the patients in the training dataset. The proportion of females to males was set to 0.5 as there was no mention of gender in the clinical study. All simulations run for 10 groups of 10 persons each to study the population PK. The virtual study ran for 168 h. Patients received 60 mg/m^2^ of DOX via IV bolus infusion over 2 min at time 0 in accordance with the conditions of the clinical study. Finally, we opted to record 10,000 virtual plasma samples over the course of 168 h.

### 2.6. Results Based on Training Dataset

**Figure 2:**
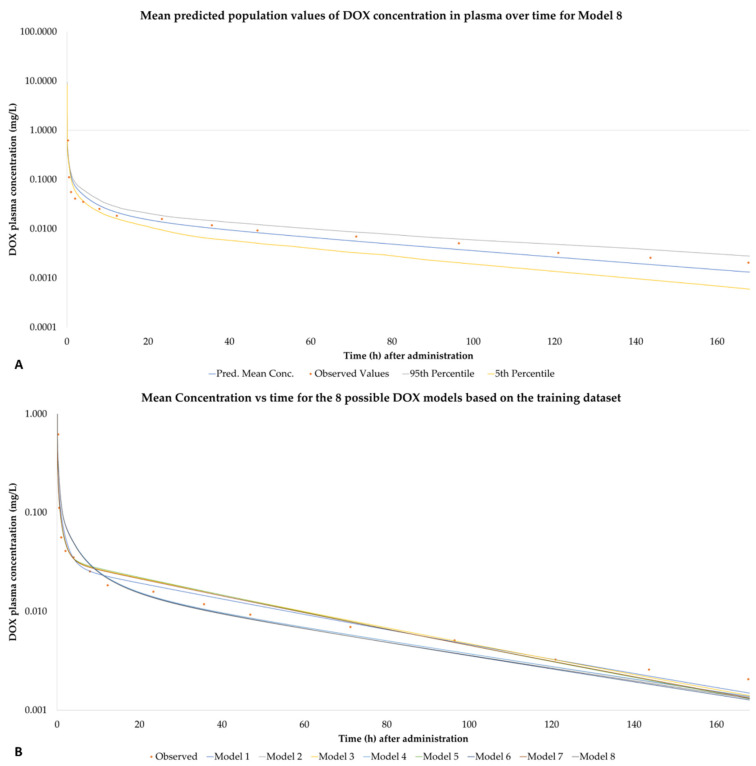
(**A**) Mean, 95th percentile and 5th percentile of the concentration versus time of DOX for model 8 based on the works of Camaggi et al. DOX was given as a single IV bolus injection of 60 mg/m^2^ at 0 h. (**B**) Comparative mean concentration vs. time for all 8 DOX models.

Figure 2A represents the predicted mean, 95th percentile and 5th percentile concentration versus time course of DOX for model 8, presented as a sample, for the virtual patient population. Figure 2B shows the mean concentration vs. time values for all 8 models. For the plots of the remaining seven models, see Supplementary Materials Section S13.

### 2.7. Observed Concentration Values for the Trainind Dataset

Table 8 summarizes the AUC and C_max_ for the different models. The observed mean value for AUC_0–168_ was reported by the authors in their clinical study and was calculated after the exclusion by us of two patients (see Section 2.4). The predicted population mean AUC_0–168_ for each model was calculated by the Simcyp simulator. The observed mean C_max_ value was calculated for time 0 h based on the triexponential equations the authors of the clinical study suggested for DOX resulting from their measurements. The predicted mean C_max_ of the population for each model was calculated by the simulator. 

## 3. Results-Validation of Models

### 3.1. Patient Demographics for the Development of the Model Based on the Validation Dataset

As mentioned earlier, the clinical study of Speth et al. was used as the validation dataset. In their work, Speth et al. studied the pharmacokinetic behaviour of DOX, by analysing its concentration both in plasma and at a cellular level [23]. Eighteen patients with leukaemia participated in the study. Their age ranged from 17 to 67 y.o. and there were 8 females and 10 males. All patients had normal renal and hepatic function.

### 3.2. Administration and Sample Retrieval

The patients were administered Vincristine on day 2 (dose of 1 mg/m^2^), and Cytarabine each day for days 1 to 7 (dose 200 mg/m^2^). In sixteen patients, DOX was administered on days 1, 2 and 3 (dose of 30 mg/m^2^) and seven patients received DOX as an IV bolus injection, five as a 4 h IV infusion, four as an 8 h IV infusion. In two patients, DOX was administered on day 1 as a 72 h IV infusion. Blood samples were taken from 5 to 240 min after administration from at least 2 patients per therapeutic scheme. For the rest of the patients in each therapeutic scheme, blood samples were taken when DOX is expected to reach its maximum and minimum concentration. After centrifuging for 10 min, plasma was kept at −20 °C. Two of the therapeutic schemes were selected for independent validation of the models. Specifically, the IV bolus and the 8 h IV infusion of DOX.

### 3.3. Analytical Method and Pharmacokinetic Analysis

The samples were analysed via high-performance liquid chromatography (HPLC). The sensitivity of the analytical methodology was 1 ng/mL. DOX plasma concentrations were described by biexponential equations. C_max_ values (at 5 min after the third administration) and AUC for each therapeutic scheme are summarized in Table 9. 

### 3.4. Clinical Settings of Virtual Patients for Validation Dataset

Again, the simulated cancer population was selected for the simulation, as it better represents the clinical study population of Speth et al., used as a validation dataset. As the Simcyp simulator requires a minimum age of 20 for this specific population, the age of the virtual patients was set from 20 to 67 years. The female analogy was set at 44.5% in accordance with the study. Ten groups of 10 patients each were simulated. The study duration was set at 168 h. The dose was set at 30 mg/m^2^, based on the selected therapeutics schemes.

### 3.5. PBPK Models Performance

**Figure 3:**
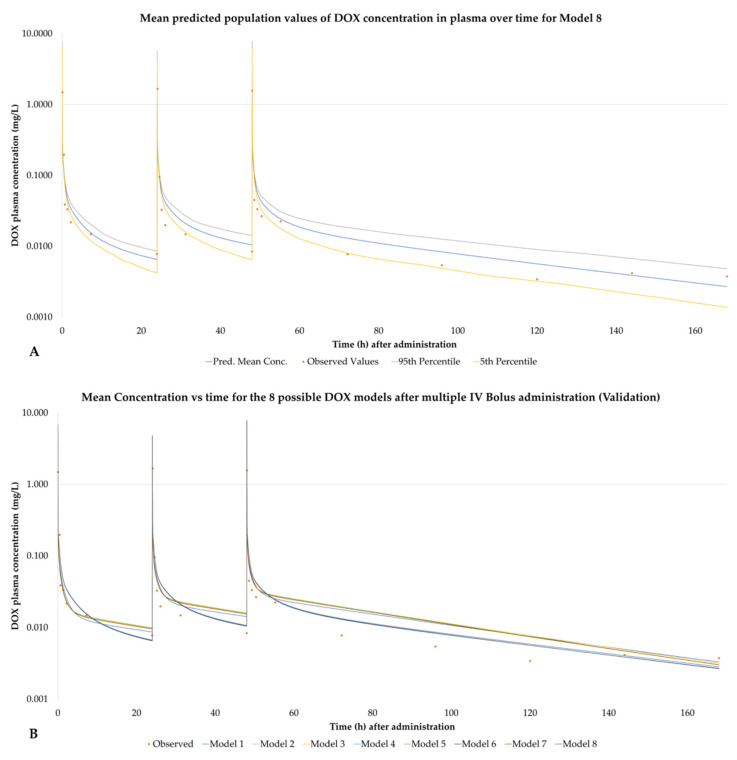
(**A**) Mean, 95th percentile and 5th percentile of the concentration versus time of DOX for model 8 based on the works of Speth et al. DOX was given as a 3-day IV bolus injection of 30 mg/m^2^ every 24 h. (**B**) Comparative mean concentration vs. time for all 8 DOX models for the above-mentioned administration.

Figure 3A represents the predicted mean, 95th percentile and 5th percentile concentration versus time course of DOX for model 8, presented as a sample, for the virtual patient population for the IV bolus injection trial (administration of 30 mg/m^2^ on days 1, 2 and 3). Figure 3B shows the mean concentration vs. time values for all 8 models. For the plots of the remaining seven models, see Supplementary Materials Section S14.

**Figure 4:**
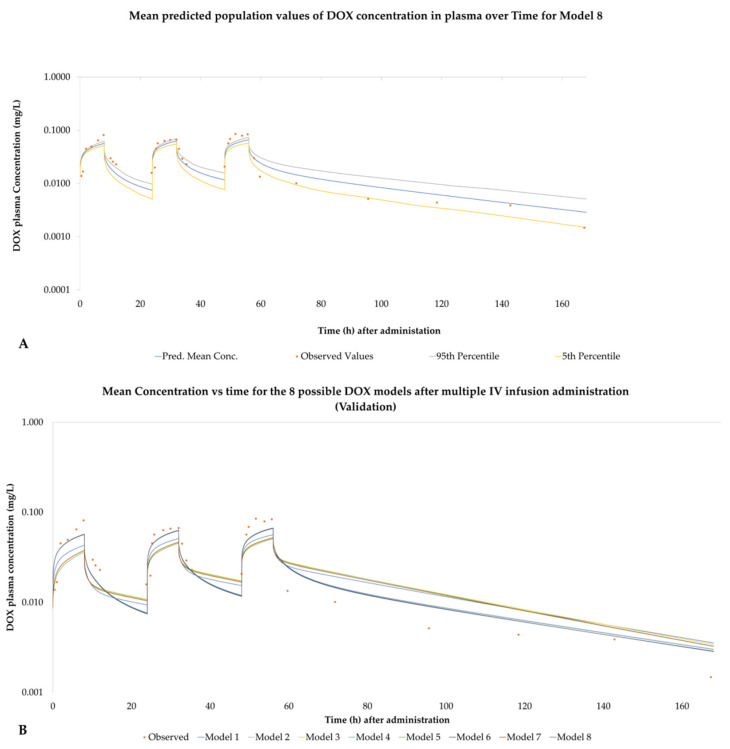
(**A**) Mean, 95th percentile and 5th percentile of the concentration versus time of DOX for model 8 based on the works of Speth et al. DOX was given as a 3-day IV Infusion over 8 h of 30 mg/m^2^ every 24 h. (**B**) Comparative mean concentration vs. time for all 8 DOX models for the above-mentioned administration.

Figure 4A represents the predicted mean, 95th percentile and 5th percentile concentration versus time course of DOX for model 8, presented as a sample, for the virtual patient population for the DOX IV infusion trial (administration over 8 h of 30 mg/m^2^ on days 1, 2 and 3). Figure 4B shows the mean concentration vs. time values for all 8 models. For the plots of the remaining seven models, see Supplementary Materials Section S15.

### 3.6. Observed Concentration Values for the Validation Dataset

Table 10 and Table 11 summarize the AUC and C_max_ values (observed vs. predicted) for the eight different models for the two validation trials (IV bolus and IV infusion accordingly). 

The observed mean values for AUC_0–120_ were reported by the authors in their clinical study. The predicted population mean for AUC_0–120_ for each model was calculated by the simulator. This applies to both validation approaches (IV bolus and IV infusion).

In the multiple IV bolus administration, the authors of the clinical study used as the validation dataset reported for DOX, a t_1/2_ a (distribution half-life) of 4 ± 2 min. The injection was administered over 1 min. However, the first sampling time was 5 min. Based on these facts, we believe that if the biexponential equations the authors suggested based on their observations are used for the calculation of C_max_ values, then they would be underestimated. To this end, and since as noted in the study, the authors measured the concentration of DOX for one patient every 30 s after the administration and found the C_max_ value to be 9.98 mg/L at 90 s, we elected to use this value as the observed C_max_ for the multiple IV bolus administration, as we consider it to be more accurate. The predicted C_max_ values were calculated by the simulator. In the multiple IV infusion administration, the mean observed C_max_ value is directly reported by the authors. The predicted C_max_ values were calculated by the simulator.

## 4. Discussion

To accomplish the goals, the development of all DOX models followed the middle-out approach as presented above. The data regarding DOX physicochemical properties were collected from the literature or calculated, and two independent clinical studies were selected. The first served as the training dataset (clinical study by Camaggi et al.) and the second as the validation dataset (clinical study by Speth et al.) for the models. In particular, the training dataset was used for the calculation of DOX clearance. DOX distribution was calculated using data from the training dataset as well as utilizing the Simcyp parameter estimation tool (P.E. tool). The validation dataset was used to validate the models’ performance. There were three key points where different approaches could be used, thus leading to eight different possible models each of which was validated against the validation dataset. The validation was performed comparing the observed versus the predicted values for AUC and C_max_ of DOX. 

### 4.1. Discussion of the Performance of the Different DOX Models

#### 4.1.1. Model Performance Based on Training Dataset

Based on values from Table 8, the best performance based on C_max_ was observed on models 3 and 7 [−9.3% and −9.4% (relative difference between predicted and observed values), respectively] followed by that of models 1 and 5 (+11.2% and −16.3%, respectively). It seems that models based on the minimal PBPK distribution model better predict C_max_, while models using the full PBPK model seem to overestimate C_max_ by approximately 60%. This is an intriguing result; however, by considering that: (a) in the clinical case study used as the training dataset the authors measured the first concentration value at 15 min, (b) the observed C_max_ value was calculated based on the triexponential equations the authors calculated and (c) the first half-life of DOX is approximately 5 min, then one could assume that the authors probably had underestimated C_max_. 

The best-performing models when using AUC_0–168_ as the criterion seem to be models 4, 2, 7 and 5 (+0.07%, +0.16%, −0.24% and +0.56%, respectively), without significant differences for the rest. The results were satisfying, ranging from −1.79% to +1.9%. Overall, as expected, the generated DOX models fit the data of the training dataset.

#### 4.1.2. Model Performance Based on IV Bolus Validation

The first validation of the models was performed using the IV bolus methodology described in the validation dataset clinical study. As seen in Table 10, when using C_max_ as the criterion, the best performing models are 2, 4, 6 and 8 (−30.9%, −30.9%, −31.0% and −31.0%, respectively). In fact, it seems that the most accurate models are those using full PBPK model for distribution. It must be noted, however, that the observed C_max_ value corresponds to one patient whose plasma DOX concentration was measured at 90 s after administration, while the predicted values correspond to approximately 60 s after administration. For that reason, we elected to utilise the AUC predicted/observed ratio for the selection of the most appropriate model.

Utilizing AUC_0–120_ as the criterion, models 8, 7, 5 and 6 (+9.59%, +9.64%, +9.90% and +10.33%, respectively) seem to be most accurate. Among them, the best performance was by models using the approach by Wright et al. when predicting renal clearance (models 8 and 7 versus models 5 and 6). Considering all the above, it seems that the best modelling approach would be to use the full PBPK model for distribution (Method F), combined with the approach based on the work by Wright et al. when calculating renal clearance (Method B). 

#### 4.1.3. Model Performance Based on IV Infusion Validation

The second validation of the models was performed using the IV infusion methodology described in the validation dataset clinical study. As seen in Table 11, when using C_max_ as the criterion, the best-performing models are 2, 4, 6 and 8 (−59.4%, −59.5%, −59.7% and −59.7%, respectively), thus also indicating that the models using the full PBPK model for distribution tend to better predict the C_max_; however, the rest of the models show a similarly good prediction for C_max_ (values ranging from −62.0% to −65.4%).

On the other hand, using AUC_0–120_ as the criterion, the best-performing models seem to be 7 and 5 followed by 8 and 1 (24.71%, 25.08%, 25.67% and 25.91% respectively). Thus three (5,7,8) out of four best-performing models use Method B for the calculation of renal clearance, also three (1,5,7) use the mPBPK model for distribution and two of the best performing models calculate hepatic metabolic clearance by each method (C and D).

It is noteworthy, however, that the differences between observed and predicted values are relatively small and well within the two-fold ratio for acceptance set by the industry as a performance standard [30,31,32].

### 4.2. Limitations of the Models Based on Procedure

During the procedure followed for the development of the different models, there were three fundamental limitations that were knowingly ignored at this point but will be thoroughly investigated in the future. 

The first limitation was that as can be observed from the training dataset data the renal clearance of the drug exceeds the expected GFR of the patients thus indicating the participation of active procedures in renal excretion. Despite that, we calculated the renal clearance of a 20–30 y.o. male solely based on the expected GFR rate.

The second limitation is about the calculated blood to plasma ratio of DOX. The value used refers to rats. Thus, it is possible that it slightly differs in humans. The future development of our model will consider this factor as well.

The third and last limitation is the distribution of metabolic clearance into its different components. Based on qualitative descriptions, we attempted to quantify the relative percentage of each path. This will also be further investigated as we expand our models.

## 5. Conclusions

Based on the data presented in this work, all generated DOX models perform quite well according to the two-fold standard that is widely considered the acceptable measure for the performance of PBPK models.

However, depending on the relevant application where the model might provide insights (e.g., whether the prediction of plasma or specific tissue concentration is in question), one should choose the appropriate model. In the case that DOX nanoformulation pharmacokinetics is under consideration, the selection of a model utilizing full PBPK for distribution is probably most appropriate. The latter also applies if the purpose of the model is the prediction of DOX metabolites or DOX toxicokinetics as more accuracy is needed in the prediction of concentration at the tissue level. To further investigate the above-mentioned problematic PK behaviour of DOX (relating to gender among other pharmacogenomic factors), more clinical data are needed to refine and validate the model for also predicting DOX and its metabolites in various tissues. Overall, we believe that the developed models form a solid basis for further development of even more informative models expanding to new formulations and pharmacogenomic investigations for DOX.

## Figures and Tables

**Table 1:** Details of the clinical studies used for the development and validation of DOX PBPK model.

Dose (mg/m^2^)	Administration	N. ^1^	Women (%)	Age (Years)	Weight (kg)	BSA (m^2^)	Dataset	Reference
60	Single IV bolus	8	NA	57.63 ± 9.28 (42–72)	69.15 ± 14.86 (45.9–90.0)	1.72 ± 0.18 (1.40–1.92)	Training	[22]
30	Q24 h × 3 IV bolus	7	44.5% ^2^	44 ± 17 ^2^ (17–67)	NA	NA	Validation	[23]
30	Q24 h × 3 IV infusion over 8 h.	4	44.5% ^2^	44 ± 17 ^2^ (17–67)	NA	NA	Validation	[23]

^1^ Number of patients in the clinical study. ^2^ The study had a total of 18 patients that received different regiments of which only the mentioned two were selected. The percentage of women refers to the population of all clinical studies, since individual group values are not provided for each individual study.

**Table 2:** DOX parameters.

**Physicochemical Property**	**Value**	**Comments/References**
MW (g/mol)	543.51	Calculated
LogP_o:w_	1.27	[23]
Drug Type	Ampholyte	[24]
pKa 1	9.53	[22]
pKa 2	8.94	[22]
B:P Ratio	1.15	[25] see limitations section
f_u,p_	0.25	drugbank.ca (accessed on 20 December 2021) Supplementary Materials Section S2
**Elimination**	**Value (CV%)**	**Comments/References**
Renal Clearance L/h	7.04 ± 2.10 (29.8%) 8.67 ± 2.85(32.86%)	Method A Method B
Biliary excretion μL/min/10^6^ cells	24.80 ± 10.89 (43.93%)	-
Metabolic Clearance	-	-
μL/min/10^6^ cells (HEP)	30.38 ± 13.34(43.93%)	Method C
μL/min/mg protein (HLM)	86.29 ± 38.12(44.17%)	-
μL/min/mg protein (HLC)	38.74 ± 17.01(43.93%)	-
Distribution of clearance	See Section 2.4.2	Method D
**Distribution**	**Value**	**Comments/References**
Minimal PBPK	-	Method E
V_ss_ (L/kg) V_sac_ (L/kg) Q_sac_ (L/h)	31.923 P.E. tool P.E.	U.I. See Section 2.4.4 See Section 2.4.4
Full PBPK	-	Method F
V_ss_ (L/kg) K_p_ Scalar	34.831 P.E. tool	Predicted by Method 3 See Section 2.4.4

P.E. tool: parameter estimation tool (i.e., a Simcyp simulator tool); HEP: intrinsic metabolic clearance calculated per 10^6^ hepatocytes; HLM: intrinsic metabolic clearance calculated per mg of microsomal protein; HLC: intrinsic metabolic clearance calculated per mg of cytosolic protein; V_ss_: Volume of distribution in steady state; V_sac_: volume of single adjusting compartment (see Supplementary Materials Section S12 for details); Q_sac_: single adjusting compartment blood flow (see Supplementary Materials Section S12 for details); K_p_ Scalar: A scaling value for the calculated K_p_ values of each tissue in a full PBPK model (see Supplementary Materials Section S12 for details).

**Table 3:** Simcyp in vitro-in vivo scaling for hepatic clearance.

System	Prediction	*CLu_int_* Measuring Unit	Scaling Factor 1	Scaling Factor 2
Hepatocytes	Metabolic clearance Biliary excretion	μL/min per 10^6^ cells	HPGL	Liver weight
Cytosolic fraction	Metabolic clearance	μL/min per mg of protein	CPPGL	
Microsomal fraction	Metabolic clearance	μL/min per mg of protein	MPPGL	

HPGL: hepatocytes per gram liver, CPPGL: cytosolic protein per gram liver, MPPGL: microsomal protein per gram liver.

**Table 4:** Percentage of CO for liver via hepatic artery and portal vein based on gender.

Gender	Through Hepatic Artery	Through Portal Vein	Total
Males	6.5%	19.0%	25.5%
Females	6.5%	21.5%	28.0%
Average for both genders			26.75%

**Table 5:** Fractions of DOX excreted via different paths.

Way of Elimination	Percentage
*f_bile_* (%)	40%
*f_met_* (%)	49%
*f_e_* (%)	11%

**Table 6:** Relative contribution of metabolic clearance per 10^6^ hepatocytes, per mg of microsomal protein and per mg of cytoplasmic protein.

Pathway	Approximate Relative Contribution	*CL_int_*	Measuring Unit	CV %
HLM	37%	31.929	μL/min/mg	44.17%
HLC	60%	23.241	μL/min/mg	43.93%
HEP	3%	0.911	μL/min/10^6^ cells	43.93%

**Table 8:** Comparison of observed vs. predicted values of C_max_ and AUC for the 8 possible DOX models based on the training dataset.

	Parameters			C_max_ (mg/L)			AUC_0–168_ (mg∙h/L)		
Model	*CL_R_* (L/h)	*CL_met_*	Dist. *	Pred.	Obs.	Diff.	Pred.	Obs.	Diff.
1	7.04	HEP	mPBPK	6.085	5.474	11.2%	1.976	1.939	1.90%
2	7.04	HEP	fPBPK	9.048		65.3%	1.942		0.16%
3	7.04	DIST	mPBPK	4.963		−9.3%	1.970		1.59%
4	7.04	DIST	fPBPK	9.048		65.3%	1.940		0.07%
5	8.67	HEP	mPBPK	4.582		−16.3%	1.950		0.56%
6	8.67	HEP	fPBPK	9.027		64.9%	1.909		−1.56%
7	8.67	DIST	mPBPK	4.957		−9.4%	1.934		−0.24%
8	8.67	DIST	fPBPK	9.022		64.8%	1.904		−1.79%

* This term (Dist.) refers to DOX distribution model. *CL_met_*: represents the metabolic clearance for each model calculated either by 10^6^ hepatocytes (HEP) or using our custom distribution (DIST) of metabolic clearance on in vitro systems (see Table 6). For the origin of observed values, see Section 2.7.

**Table 9:** Validation dataset pharmacokinetic parameters of patients.

Therapeutic Scheme	Dose	N. of Patients	C_max_ (ng/mL)	AUC_0–120_ (mg × h/L)	V_ss_ (L)
IV bolus	30 mg/m^2^ Q24 h × 3	7	1640 ± 470 (9980 at 90 s)	2.3 ± 0.5	1450 ± 84
8 h infusion	30 mg/m^2^ Q24 h × 3	4	85 ± 50	2.0 ±1.3	

**Table 10:** Comparison of observed vs. predicted values of C_max_ and AUC_0–168_ for the 8 possible DOX models based on the validation dataset and IV bolus administration.

	Parameters			C_max_ (mg/L)			AUC_0–120_ (mg∙h/L)		
Model	*CL_R_* (L/h)	*CL_met_*	Dist. *	Pred.	Obs.	Diff.	Pred.	Obs.	Diff.
1	7.04	HEP	mPBPK	4.331	9.980	−56.6%	2.542	2.300	10.51%
2	7.04	HEP	fPBPK	6.896		−30.9%	2.574		11.89%
3	7.04	DIST	mPBPK	3.787		−62.1%	2.560		11.32%
4	7.04	DIST	fPBPK	6.896		−30.9%	2.563		11.45%
5	8.67	HEP	mPBPK	3.560		−64.3%	2.528		9.90%
6	8.67	HEP	fPBPK	6.884		−31.0%	2.538		10.33%
7	8.67	DIST	mPBPK	3.784		−62.1%	2.522		9.64%
8	8.67	DIST	fPBPK	6.882		−31.0%	2.521		9.59%

* This term refers to DOX distribution model. *CL_met_*: represents the metabolic clearance for each model calculated either by 10^6^ hepatocytes (HEP) or using our custom distribution (DIST) of metabolic clearance on in vitro systems (see Table 6).

**Table 11:** Comparison of observed vs. predicted values of C_max_ and AUC_0–168_ for the 8 possible DOX models based on the validation dataset and IV infusion over 8 h administration.

	Parameters			C_max_ (μg/L)			AUC_0–120_ (mg∙h/L)		
Model	*CL_R_* (L/h)	*CL_met_*	Dist. *	Pred.	Obs.	Diff.	Pred.	Obs.	Diff.
1	7.04	HEP	mPBPK	32.316	85.000	−62.0%	2.518	2.000	25.91%
2	7.04	HEP	fPBPK	34.521		−59.4%	2.566		28.31%
3	7.04	DIST	mPBPK	30.411		−64.2%	2.532		26.62%
4	7.04	DIST	fPBPK	34.441		−59.5%	2.556		27.79%
5	8.67	HEP	mPBPK	29.439		−65.4%	2.502		25.08%
6	8.67	HEP	fPBPK	34.223		−59.7%	2.531		26.56%
7	8.67	DIST	mPBPK	30.080		−64.6%	2.494		24.71%
8	8.67	DIST	fPBPK	34.077		−59.9%	2.513		25.67%

* This term refers to DOX distribution model. *CL_met_*: represents the metabolic clearance for each model calculated either by 10^6^ hepatocytes (HEP) or using our custom distribution (DIST) of metabolic clearance on in vitro systems (see Table 6).

## Data Availability

Data is contained within the article or Supplementary Material.

## Supplementary Materials

The following are available online at https://www.mdpi.com/article/10.3390/pharmaceutics14030541/s1. Section S1: Pharmacokinetic data of DOX from the training dataset. Section S2: Plasma Protein Binding. Section S3: Calculating Renal Clearance for each patient. Section S4: Calculating liver blood flow for each patient. Section S5: DOX blood to plasma ratio. Section S6: Hepatic Blood clearance (CLH.B) of each patient. Section S7: Calculating mean fraction excreted in urine for the patients. Section S8: Intrinsic Hepatic Clearance (*CLu_int,H,b_*), Intrinsic Biliary Excretion (*CLu_bile,b_*) and Intrinsic Metabolic Clearance (*CLu_int,met,b_*) for each patient. Section S9: Calculating Liver Weight, HPGL, MPPGL and CPPGL for each patient. Section S10: Biliary excretion per 10^6^ of patient hepatic cells. Section S11: Calculating the corrected intrinsic metabolic clearance of each patient based on the three different in vitro systems. Section S12: Explaining Simcyp distribution models. Section S13: Figures of DOX population concentration vs time for the 8 models based on the Training dataset. Section S14: Figures of DOX population concentration vs time for the 8 models based on the Validation dataset multiple IV Bolus administration. Section S15: Figures of DOX population concentration vs time for the 8 models based on the Validation dataset multiple IV Infusion administration. Table S1: Pharmacokinetic data of DOX from the training dataset. Table S2: Calculated Renal clearance values based on the work of Davies and Shock for each patient. Table S3: Calculated Renal clearance values based on the work of Wright et al. for each patient. Table S4: Calculated liver blood flow values for each patient. Table S5: Calculated hepatic blood clearance values for each patient. Table S6: Calculated mean fraction excreted in urine for each patient. Table S7: Intrinsic Hepatic Clearance (*CLu_int,H,b_*), Intrinsic Biliary Excretion (*CLu_bile,b_*) and Intrinsic Metabolic Clearance (*CLu_int,met,b_*) for each patient. Table S8: Calculated Liver Weight, HPGL, MPPGL and CPPGL for each patient. Table S9: Calculated biliary excretion per 10^6^ of patient hepatic cells. Table S10: Calculated corrected intrinsic metabolic clearance of each patient based on the three different in vitro systems. Figures S1–S7: Mean, 95th percentile and 5th percentile of the concentration versus time of DOX for models 1–7 based on the works of Camaggi et al. DOX was given as a single IV bolus injection of 60 mg/m^2^ at 0 h. Figures S8–S14: Mean, 95th percentile and 5th percentile of the concentration versus time of DOX for models 1–7 based on the works of Speth et al. DOX was given as a 3-day IV bolus injection of 30 mg/m^2^ every 24 h. Figures S15–S21: Mean, 95th percentile and 5th percentile of the concentration versus time of DOX for model 1–7 based on the works of Speth et al. DOX was given as a 3-day IV Infusion over 8 h of 30 mg/m^2^ every 24 h. (References [22,28,29,33,34,35] are cited in the Supplementary Materials).
