# Supplementary material for: Physiologically Based Pharmacokinetic Modelling and Simulation to Predict the Plasma Concentration Profile of Doxorubicin
Source: Pharmaceutics. 2022 Feb 28;14(3):541. doi: 10.3390/pharmaceutics14030541 (PMC8949582; doi:10.3390/pharmaceutics14030541)
Supplement: Supplementary file 1 [file pharmaceutics-14-00541-s001.zip › pharmaceutics-1574636-supplementary.pdf]

pharmaceutics

Supplementary Material

Physiologically Based Pharmacokinetic Modelling and Simulation to Predict the Plasma Concentration Profile of Doxorubicin

George A. Mystridis [1], Georgios C. Batzias [2] and Ioannis S. Vizirianakis [1,3,*]

[1] Laboratory of Pharmacology, School of Pharmacy, Faculty of Health Sciences, Aristotle University of Thessaloniki, GR-54124 Thessaloniki, Greece; geormyst@pharm.auth.gr
[2] Laboratory of Pharmacology, School of Veterinary Medicine, Faculty of Health Sciences, Aristotle University of Thessaloniki, GR-54124 Thessaloniki, Greece; batzias@vet.auth.gr
[3] Department of Life and Health Sciences, School of Sciences and Engineering, University of Nicosia, Nicosia CY-1700, Cyprus
* Correspondence: ivizir@pharm.auth.gr

Section S1: Pharmacokinetic Data of DOX from the Training Dataset (Clinical Study by Camaggi et al. [1])

Table S1. Pharmacokinetic data of DOX from the training dataset (Clinical study by Camaggi et al.).

ID	Weight	Age	BSA	AUC (mg·h/L)	AUC_{0-168} (ng·h/L)	CL_p (L/h)	CL_R (L/h)	V_{ss} (L)	V_{ss} (L/Kg)
66	71.50	52.00	1.76	2.57	2497.70	40.87	3.94	1527.24	21.36
67	66.00	58.00	1.74	1.87	1771.90	53.53	6.80	2370.06	35.91
68	74.40	68.00	1.72	1.82	1683.10	54.94	6.62	2650.13	35.62
70	45.90	72.00	1.40	2.68	2486.20	33.62	4.90	1626.70	35.44
71	80.40	42.00	1.92	1.94	1815.70	59.26	4.29	2542.25	31.62
73	75.00	57.00	1.80	1.46	1378.40	68.29	6.97	2369.25	31.59
			$\bar{x}$	**2.06**	**1938.83**	**51.75**	**5.59**	**2180.94**	**31.923**
			**s**	**0.43**	**415.22**	**11.48**	**1.25**	**439.02**	**5.062**
			**cv**	**21%**	**21%**	**22%**	**22%**	**20%**	**15.9%**

Section S2: Plasma Protein Binding

According to Drugbank.ca DOX is 74–76% bound to plasma proteins. Thus, the unbound fraction. $f_{u.p.}$ was calculated as 24–26%. Simcyp calculated the $f_{u.p.}$ to be approximately 74.3%. so the final value selected was the average of the reported values in Drugbank. i.e., $f_{u.p}$ = 0.25.

Section S3: Calculating Renal Clearance for Each Patient

3.1. Renal Clearance Values Based on the Work of Davies and Shock

In the following table are the calculated GFR values (cGFR) for each patient as well as the adjusted Renal Clearance (aCL_R) that would correspond to a 20–30 y.o. healthy male based on the work of Davies and Shock.

Table S2. Calculated Renal clearance values based on the work of Davies and Shock for each patient.

ID	Weight (kg)	Age (years)	BSA (m^2)	CL_R (L/h)	GFR_g (mL/min/1.73m^2)	cGFR (mL/min)	αCL_R (L/h)
66	71.5	52	1.76	3.94	99.3	101.0	4.79
67	66.0	58	1.74	6.80	99.3	99.9	8.36
68	74.4	68	1.72	6.62	96.0	95.4	8.52
70	45.9	72	1.40	4.90	89.0	72.0	8.35
71	80.4	42	1.92	4.29	121.2	134.5	3.92
73	75.0	57	1.80	6.97	99.3	103.3	8.28
						$\bar{x}$	**7.04**
						**s**	**2.10**
						**cv**	**29.8%**

3.2. Renal Clearance Values Based on the Work of Wright et al.

In the following table are the calculated GFR values (cGFR) for each patient as well as the adjusted Renal Clearance (aCL_R) that would correspond to a 20–30 y.o. healthy male based on the work of Wright et al. The reference GFR value for a 20–30 y.o. male came from the work of Davies and Shock. The values were calculated as average assuming both male and female gender.

Table S3. Calculated Renal clearance values based on the work of Wright et al. for each patient.

ID	Weight (kg)	Age (Years)	BSA (m^2)	CL_R (L/h)	SCr Male	SCr Female	cGFR (mL/min) Male	cGFR (mL/min) Female	cGFR (mL/min) Average	αCL_R (L/h)
66	71.5	52	1.76	3.94	87.8	77.5	91.5	86.2	88.8	5.45
67	66.0	58	1.74	6.80	87.8	77.5	85.8	80.9	83.3	10.02
68	74.4	68	1.72	6.62	95.7	77.5	70.8	72.8	71.8	11.32
70	45.9	72	1.40	4.90	95.7	77.5	55.4	56.9	56.1	10.72
71	80.4	42	1.92	4.29	87.8	68	108.3	116.3	112.3	4.69
73	75.0	57	1.80	6.97	87.8	77.5	89.6	84.4	87.0	9.84
									$\bar{x}$	**8.67**
									**s**	**2.85**
									**cv**	**32.86%**

Section S4: Calculating Liver Blood Flow for Each Patient

In the following table are the calculated values of Liver Blood flow for each patient.

Table S4. Calculated liver blood flow values for each patient.

ID	Age (Years)	BSA (m^2)	CO (L/h)	Q_H (L/h)
66	52	1.76	283.0	75.70
67	58	1.74	273.5	73.17
68	68	1.72	260.1	69.57
70	72	1.40	208.3	55.73
71	42	1.92	320.3	85.67
73	57	1.80	284.0	75.98

Section S5: DOX Blood to Plasma Ratio

In their work Pawar et al. found that the plasma to blood plasma-to-blood ratio (P:B) was 0.870 ± 0.018 in rats [2]. Thus the blood-to-plasma ratio (B:P) was calculated to be 1.15 ± 0.02. Keeping in mind that this is a value for Rats the B:P ratio that was input into the model was 1.15.

Section S6: Hepatic Blood Clearance ($CL_{H.B}$) of Each Patient

In the following table are the calculated values of Hepatic Blood Clearance for each patient.

Table S5. Calculated hepatic blood clearance values for each patient.

ID	$CL_{H.B}$ (L/h)
66	32.11
67	40.63
68	42.02
70	24.97
71	47.80
73	53.32

Section S7: Calculating Mean Fraction Excreted in Urine for the Patients

Table S6. Calculated mean fraction excreted in urine for each patient.

ID	CL_P (L/h)	$CL_{R,P}$ (L/h)	f_e (CL_R/CL_P)
66	40.87	3.94	9.64%
67	53.53	6.80	12.70%
68	54.94	6.62	12.05%
70	33.62	4.90	14.57%
71	59.26	4.29	7.24%
73	68.29	6.97	10.21%
$\bar{x}$	**51.75**	**5.59**	**11.07%**
s	**12.57**	**1.37**	**2.58%**
cv	**24.29%**	**24.44%**	**25.66%**

Section S8: Intrinsic Hepatic Clearance ($CLu_{int,H,b}$), Intrinsic Biliary Excretion ($CLu_{bile,b}$) and Intrinsic Metabolic Clearance ($CLu_{int,met,b}$) for Each Patient

Table S7. Intrinsic Hepatic Clearance (CLuint,H,b), Intrinsic Biliary Excretion (CLubile,b) and Intrinsic Metabolic Clearance (CLuint,met,b) for each patient.

ID	$CLu_{int,H,b}$ (L/h)	$CLu_{int,bil,b}$ (L/h)	$CLu_{int,met,b}$ (L/h)
66	256.54	102.62	153.93
67	420.38	168.15	252.23
68	488.06	195.22	292.84
70	208.18	83.27	124.91
71	497.43	198.97	298.46
73	822.48	328.99	493.49

Section S9: Calculating Liver Weight, HPGL, MPPGL and CPPGL for Each Patient

Based on the work by Johnsson et al. [3] the Liver Volume (LV) depends of age and Body Surface Area according to equation:

$$LV_i = 0.722 \times BSA^{1.176} \quad (S1)$$

The mean Liver Density (LivD) has been calculated by Heinemann et al. [4] to be 1.08 g/ml. Thus, the Liver Weight (LW) for each patient can be calculated by multiplying LV and LivD:

$$LW_i = LV_i \times LivD = 0.722\ X\ BSA^{1.176}\ X\ 1.08 = 0.77976 \times BSA^{1.176} \quad (S2)$$

Simcyp simulator calculated the values for HPGL and MPPGL based on two articles by Baxter et al. [5,6]. Also it can calculate the values for CPPGL using a proprietary equation.

Table S8. Calculated Liver Weight, HPGL, MPPGL and CPPGL for each patient.

ID	LW (g)	HPGL (10^6 cells/g Liver)	MPPGL (mg protein/g Liver)	CPPGL (mg protein/g Liver)
66	1516	92.72	33.68	73.02
67	1496	89.50	31.54	70.22
68	1476	85.01	28.69	66.49
70	1158	83.45	27.93	65.50
71	1679	99.36	37.26	77.71
73	1557	90.01	31.88	70.67

Section S10: Biliary Excretion per 10^6 of Patient Hepatic Cells

Table S9. Calculated biliary excretion per 10^6 of patient hepatic cells.

ID	$CLu_{int(Bile)}$ μL/min/10^6 cells
66	13.67
67	23.52
68	29.15
70	16.13
71	22.33
73	43.97

Section S11: Calculating the Corrected Intrinsic Metabolic Clearance of Each Patient Based on the Three Different In Vitro Systems

Table S10. Calculated corrected intrinsic metabolic clearance of each patient based on the three different in vitro systems.

ID	$CLu_{int(met,HLM)}$ μL/min/mg protein	$CLu_{int(met,HLC)}$ μL/min/mg protein	$CLu_{int(met,HEP)}$ μL/min/10^6 cells
66	46.11	21.27	16.75
67	81.77	36.73	28.82
68	105.80	45.65	35.70
70	59.04	25.18	19.76
71	72.95	34.98	27.36
73	152.09	68.61	53.87
$\bar{x}$	86.29	38.74	30.38
s	38.12	17.01	13.34
cv	44.17%	43.93%	43.93%

Section S12: Explaining Simcyp Distribution Models

Two physiologically based pharmacokinetic (PBPK) models for distribution are available within Simcyp Simulator, a minimal PBPK (mPBPK) model and a full-PBPK (fPBPK) model, used to simulate concentrations in different organ compartments.

The mPBPK model is a "lumped" model with four compartments predicting only the systemic, portal vein and liver concentration. In this model the concept of Single Adjusting Compartment (SAC) is used. SAC represents a non-physiological compartment which permits adjustment to accommodate the larger volumes of distribution of heavily distributed drugs. To define it one needs two or three parameters: the volume of the SAC (V_{sac}), the input rate constant (k_{in}) and the output rate constant (k_{out}). The flow rate to SAC can be described by two rate constants if the drug distribution is permeability-limited or can be described by a single parameter if it is perfusion-limited, Q_h.

The fPBPK model is comprised of many tissues and organs each of which has its own distribution constant K_t. There is one global value ($K_{p,scalar}$) that can be used to relatively scale all other distribution constants in order to match the observed volume of distribution (V_{ss}) values.

All the above-mentioned parameters of SAC in the mPBPK model and the $K_{p,scalar}$ in the fPBPK model values can be estimated and adjusted using Simcyp Parameter Estimation (PE) tool.

Section S13: Figures of DOX Population Concentration vs. Time for the 8 Models Based on the Training Dataset

Model 1

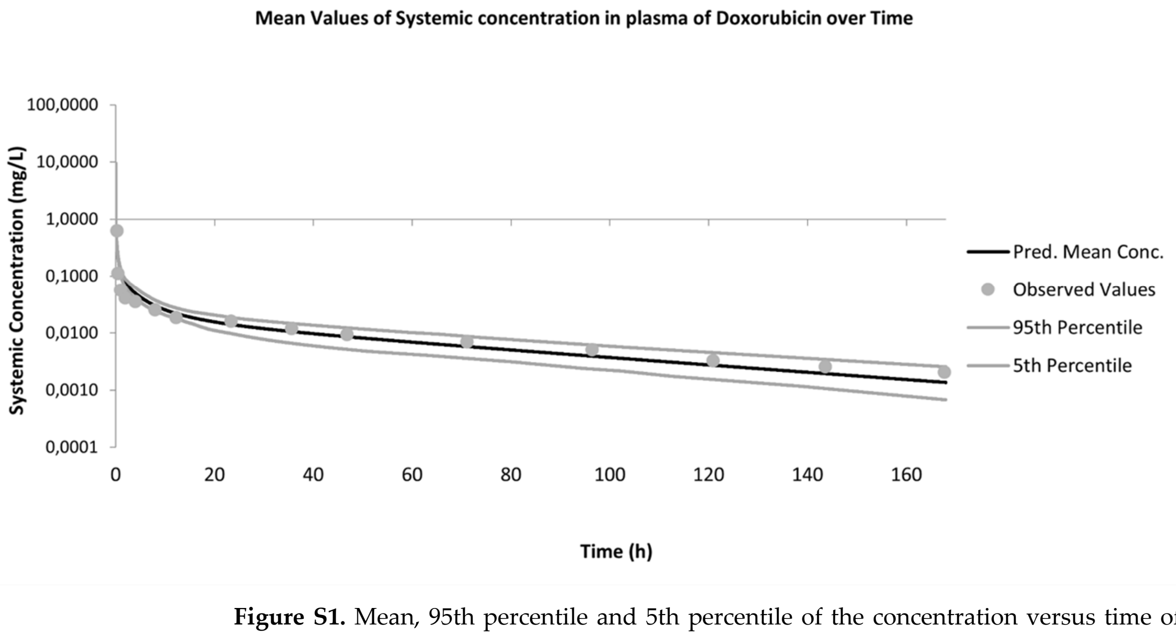

Figure S1. Mean, 95th percentile and 5th percentile of the concentration versus time of DOX for model 1 based on the works of Camaggi et al. DOX was given as a single IV bolus injection of 60 mg/m^2 at 0 h.

Model 2

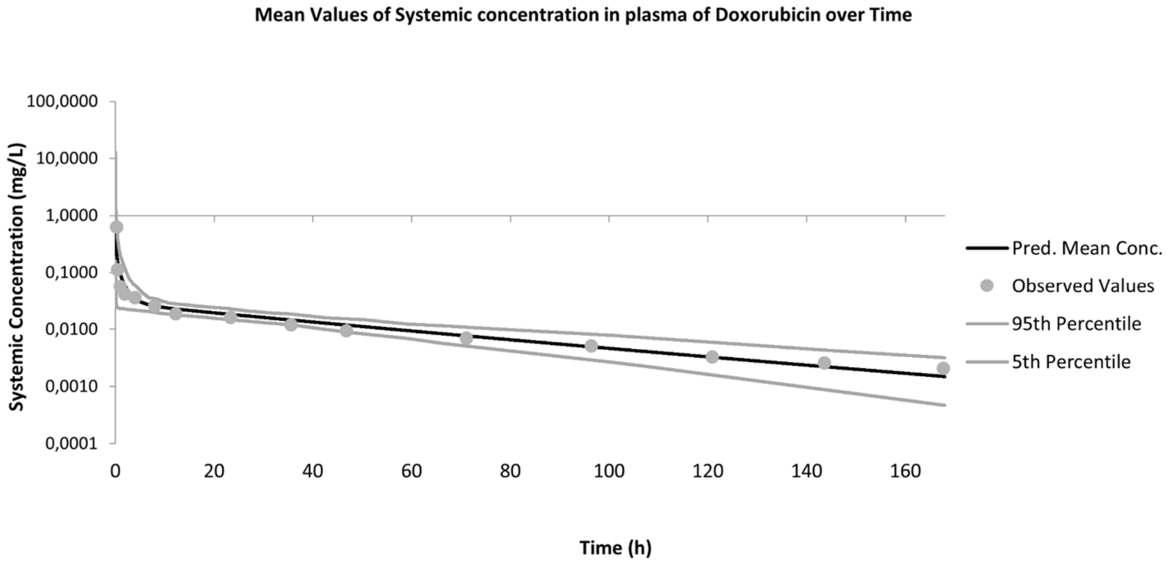

Figure S2. Mean, 95th percentile and 5th percentile of the concentration versus time of DOX for model 2 based on the works of Camaggi et al. DOX was given as a single IV bolus injection of 60 mg/m^2 at 0 h.

Model 3

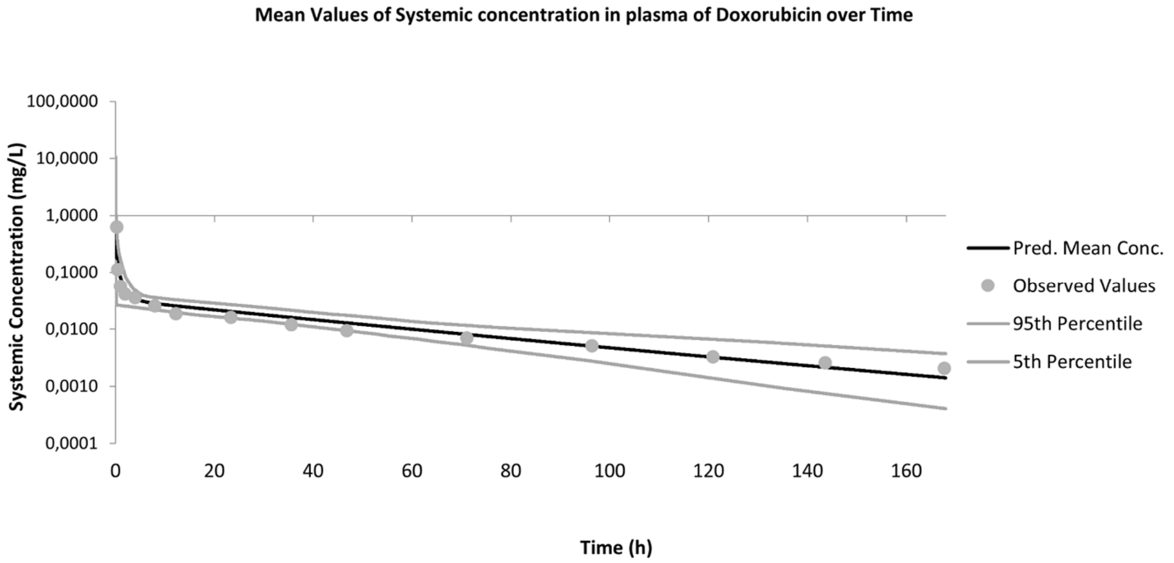

Figure S3. Mean, 95th percentile and 5th percentile of the concentration versus time of DOX for model 3 based on the works of Camaggi et al. DOX was given as a single IV bolus injection of 60 mg/m^2 at 0 h.

Model 4

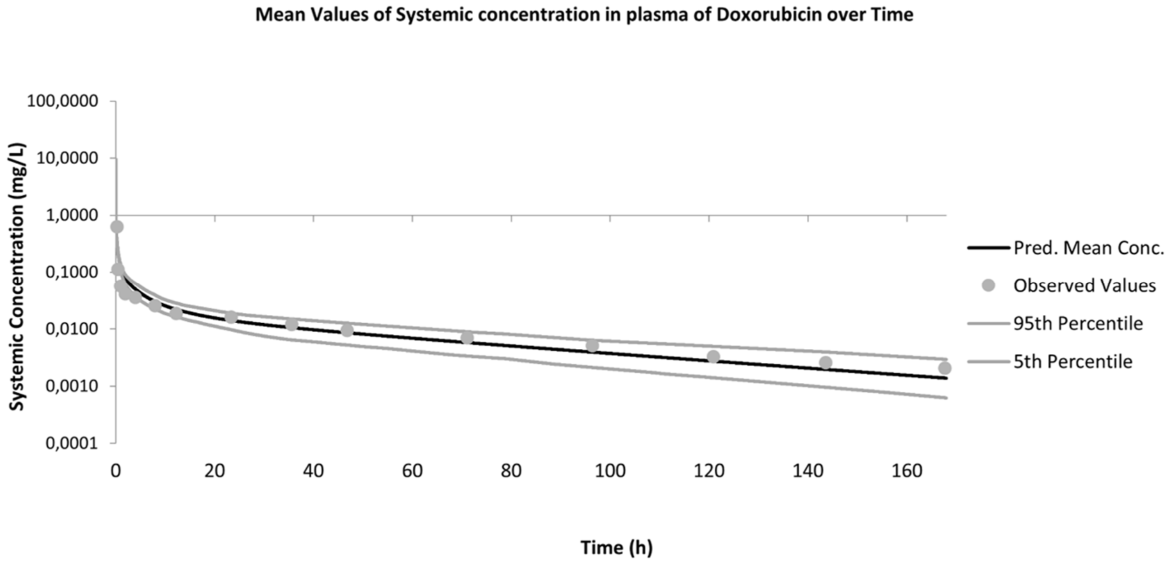

Figure S4. Mean, 95th percentile and 5th percentile of the concentration versus time of DOX for model 4 based on the works of Camaggi et al. DOX was given as a single IV bolus injection of 60 mg/m^2 at 0 h.

Model 5

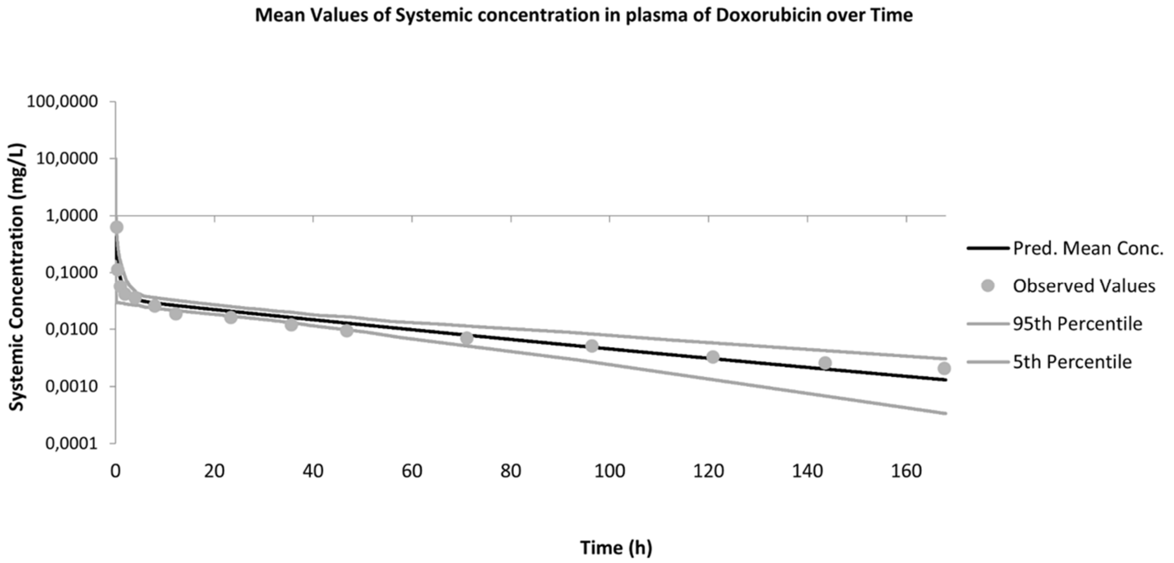

Figure S5. Mean, 95th percentile and 5th percentile of the concentration versus time of DOX for model 5 based on the works of Camaggi et al. DOX was given as a single IV bolus injection of 60 mg/m^2 at 0 h.

Model 6

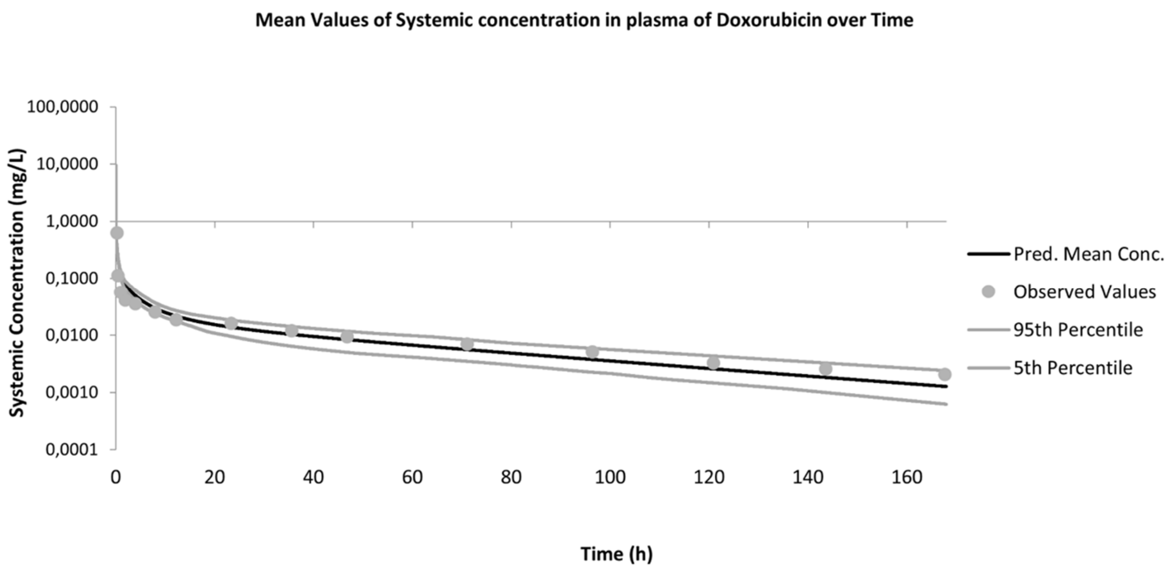

Figure S6. Mean, 95th percentile and 5th percentile of the concentration versus time of DOX for model 6 based on the works of Camaggi et al. DOX was given as a single IV bolus injection of 60 mg/m^2 at 0 h.

Model 7

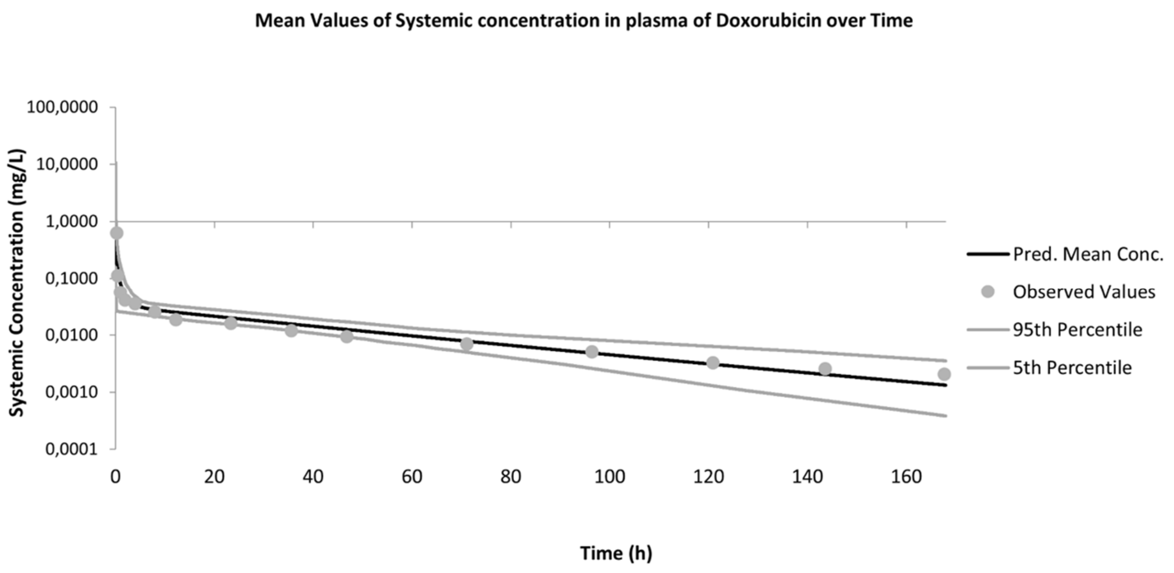

Figure S7. Mean, 95th percentile and 5th percentile of the concentration versus time of DOX for model 7 based on the works of Camaggi et al. DOX was given as a single IV bolus injection of 60 mg/m^2 at 0 h.

Section S14: Figures of DOX Population Concentration vs. Time for the 8 Models Based on the Validation Dataset Multiple IV Bolus Administration

Model 1

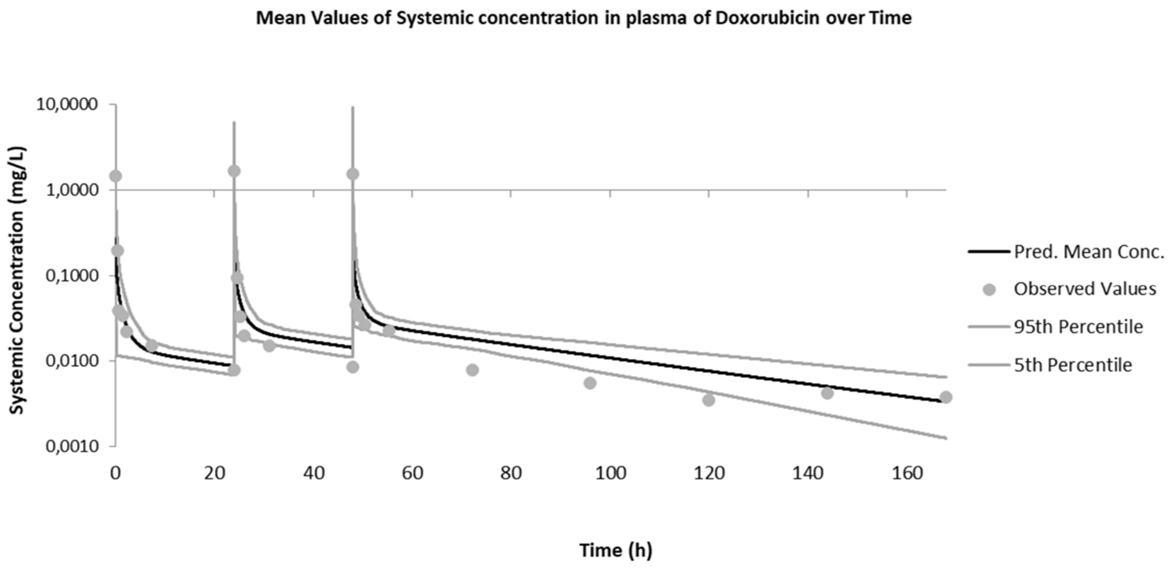

Figure S8. Mean, 95th percentile and 5th percentile of the concentration versus time of DOX for model 1 based on the works of Speth et al. DOX was given as a 3-day IV bolus injection of 30 mg/m^2 every 24 h.

Model 2

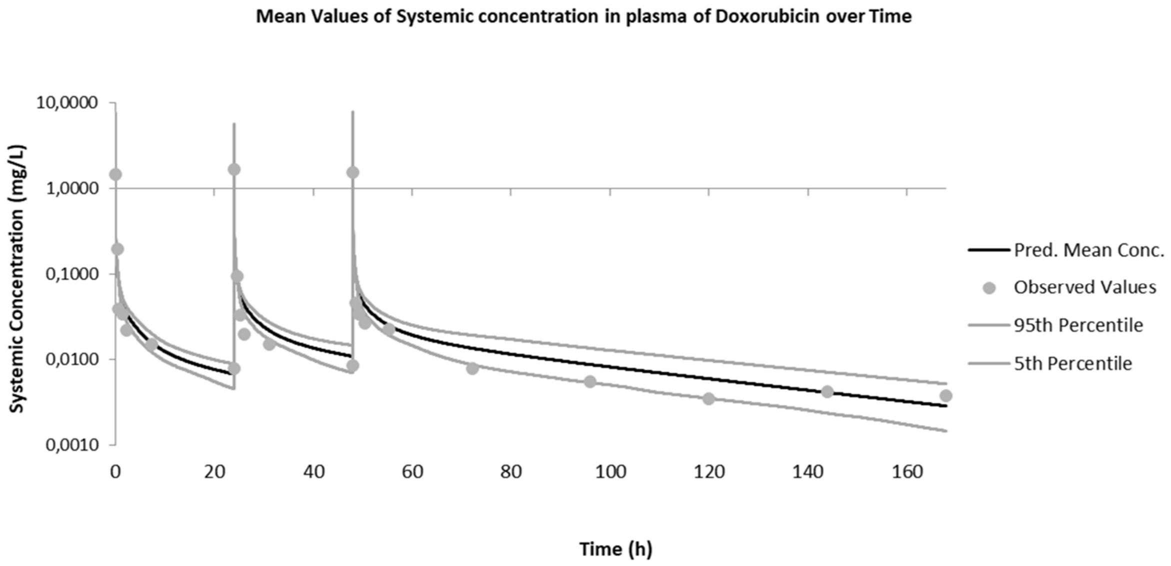

Figure S9. Mean, 95th percentile and 5th percentile of the concentration versus time of DOX for model 2 based on the works of Speth et al. DOX was given as a 3-day IV bolus injection of 30 mg/m^2 every 24 h.

Model 3

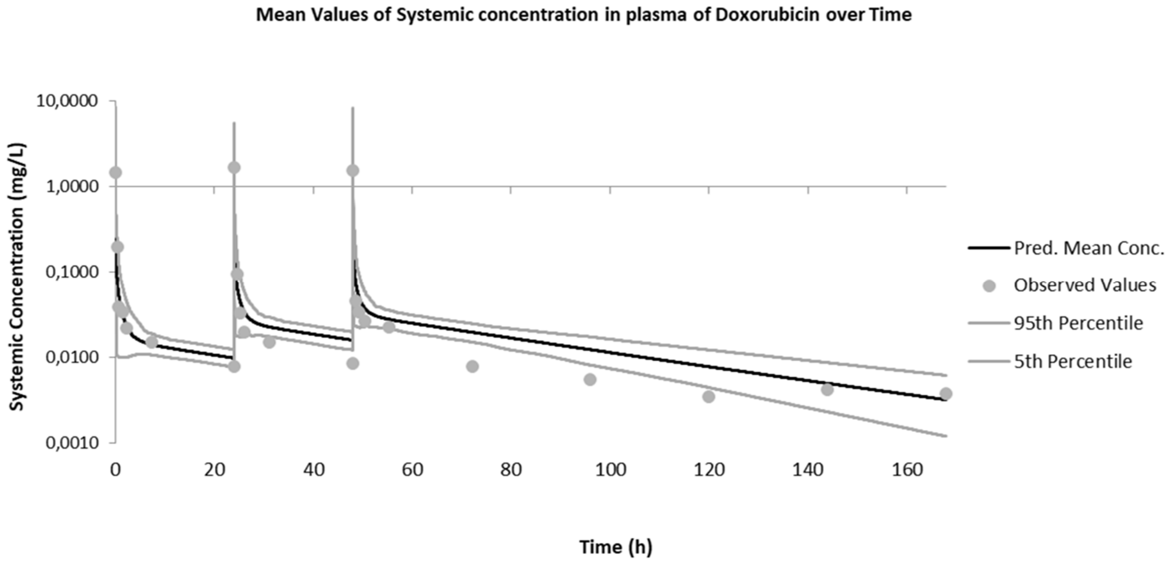

Figure S10. Mean, 95th percentile and 5th percentile of the concentration versus time of DOX for model 3 based on the works of Speth et al. DOX was given as a 3-day IV bolus injection of 30 mg/m^2 every 24 h.

Model 4

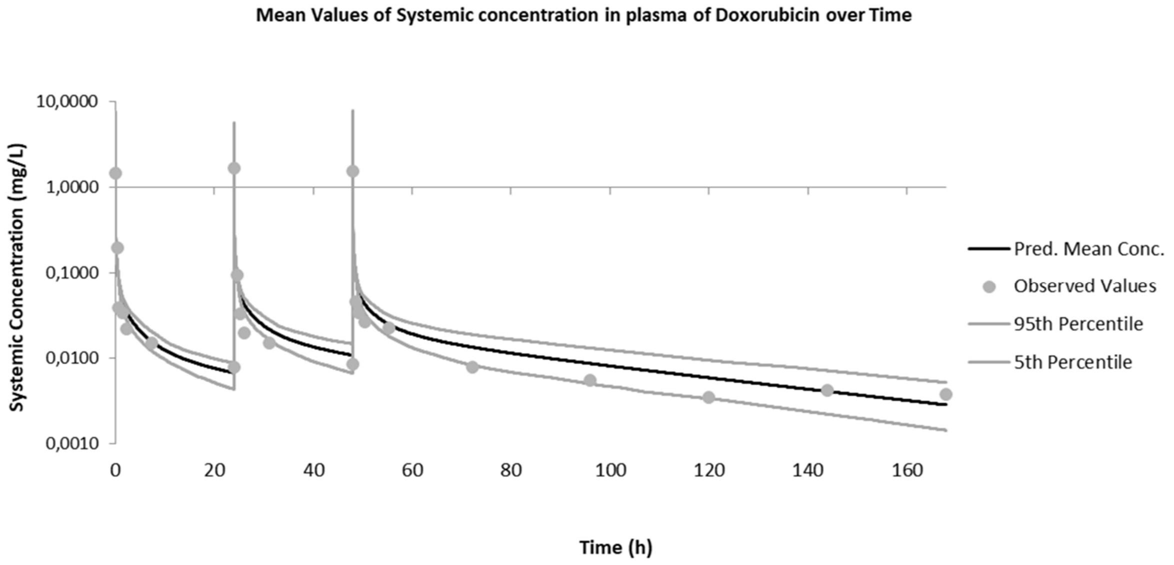

Figure S11. Mean, 95th percentile and 5th percentile of the concentration versus time of DOX for model 4 based on the works of Speth et al. DOX was given as a 3-day IV bolus injection of 30 mg/m^2 every 24 h.

Model 5

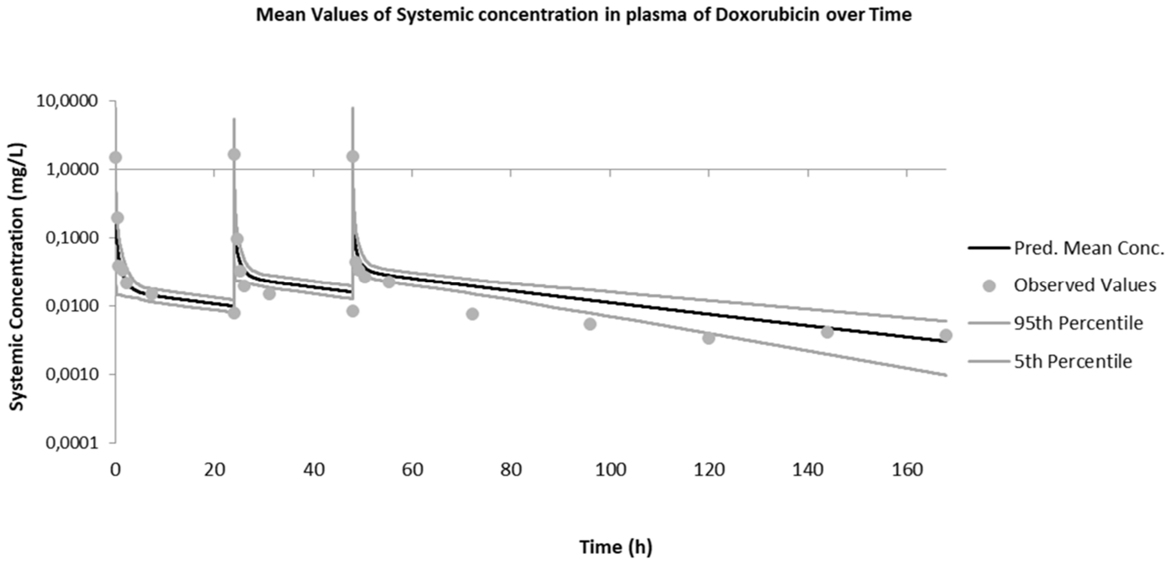

Figure S12. Mean, 95th percentile and 5th percentile of the concentration versus time of DOX for model 5 based on the works of Speth et al. DOX was given as a 3-day IV bolus injection of 30 mg/m^2 every 24 h.

Model 6

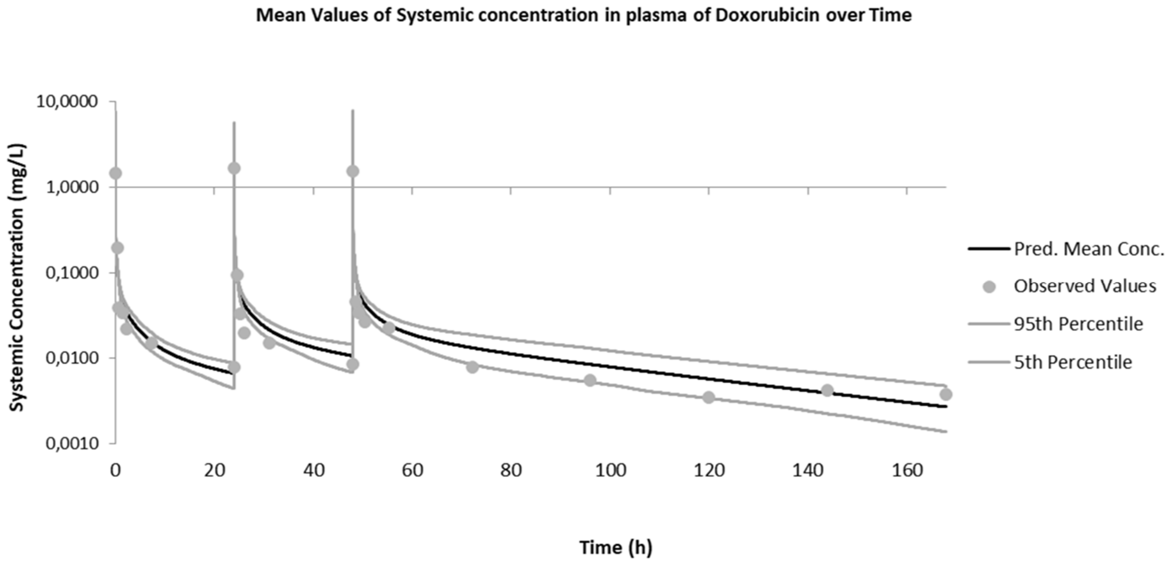

Figure S13. Mean, 95th percentile and 5th percentile of the concentration versus time of DOX for model 6 based on the works of Speth et al. DOX was given as a 3-day IV bolus injection of 30 mg/m^2 every 24 h.

Model 7

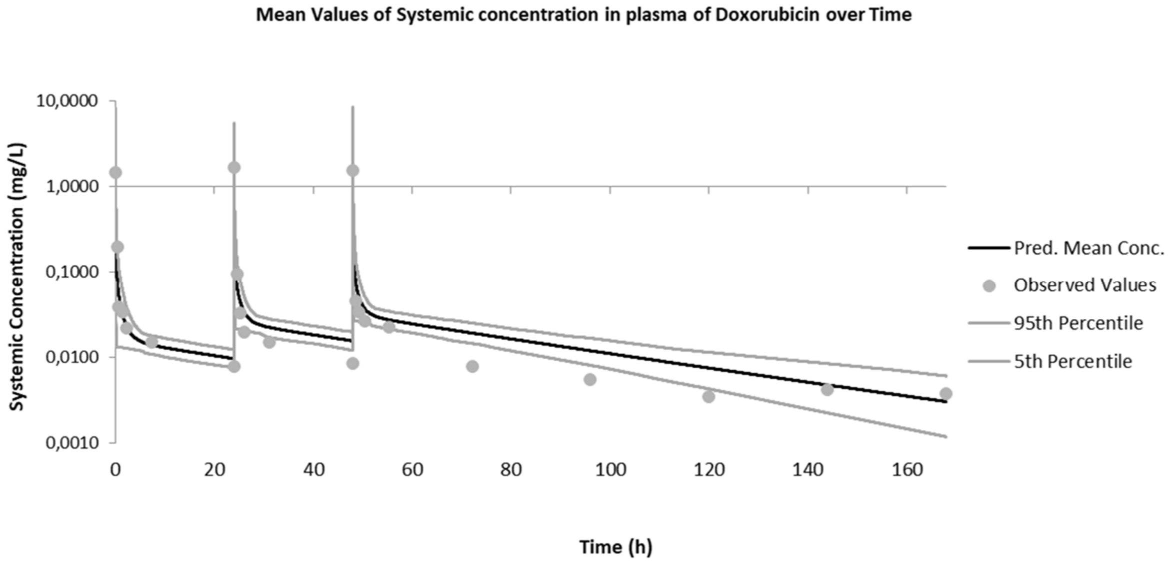

Figure S14. Mean, 95th percentile and 5th percentile of the concentration versus time of DOX for model 7 based on the works of Speth et al. DOX was given as a 3-day IV bolus injection of 30 mg/m^2 every 24 h.

Section S15: Figures of DOX Population Concentration vs. Time for the 8 Models Based on the Validation Dataset Multiple IV Infusion Administration

Model 1

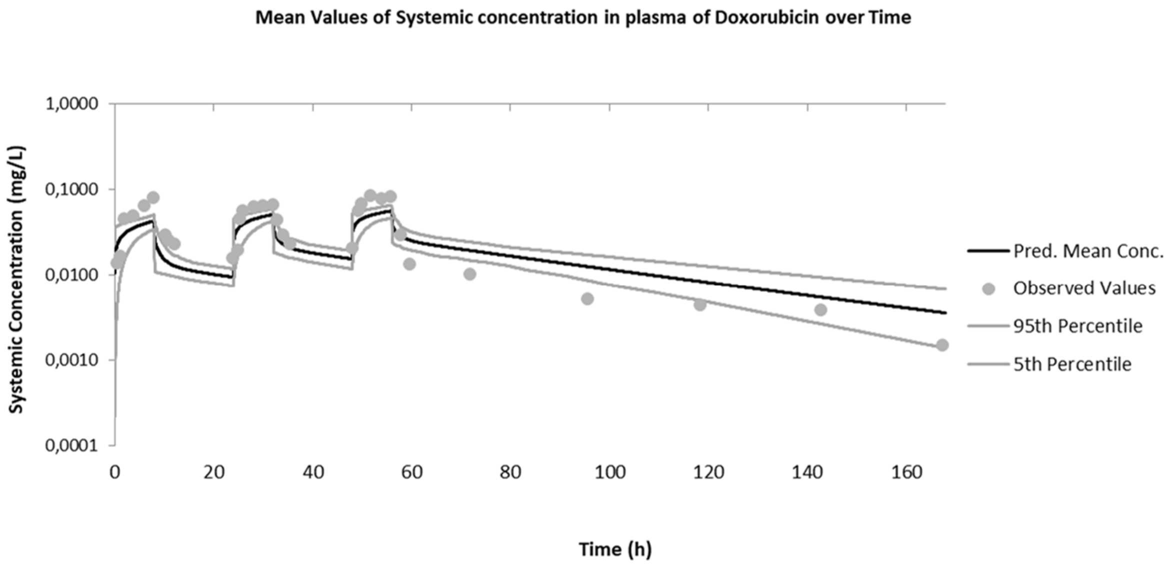

Figure S15. Mean, 95th percentile and 5th percentile of the concentration versus time of DOX for model 1 based on the works of Speth et al. DOX was given as a 3-day IV Infusion over 8 h of 30 mg/m^2 every 24 h.

Model 2

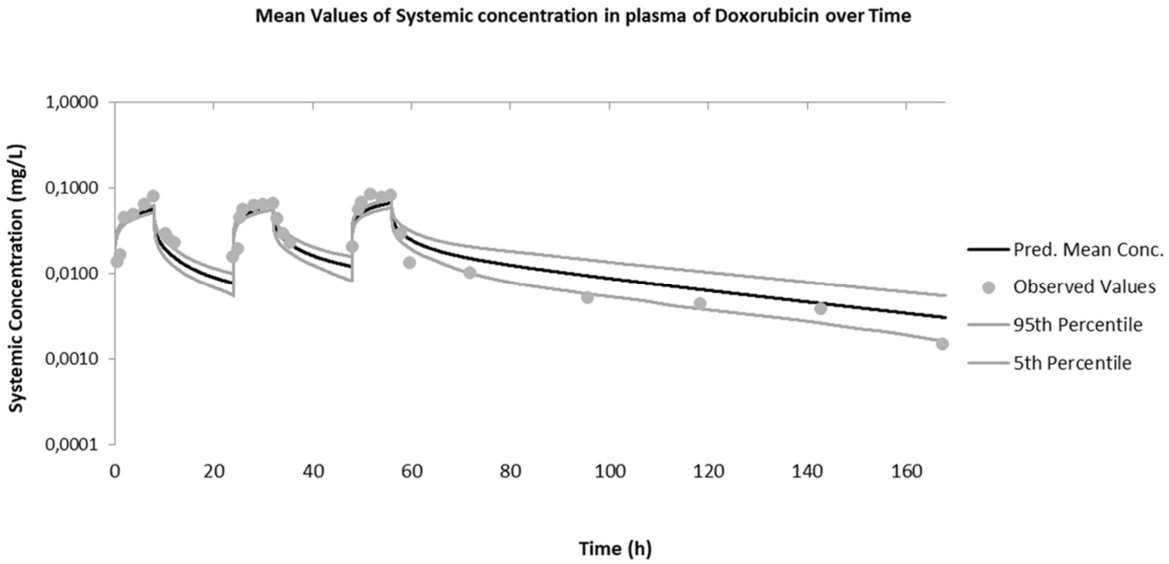

Figure S16. Mean, 95th percentile and 5th percentile of the concentration versus time of DOX for model 2 based on the works of Speth et al. DOX was given as a 3-day IV Infusion over 8 h of 30 mg/m^2 every 24 h.

Model 3

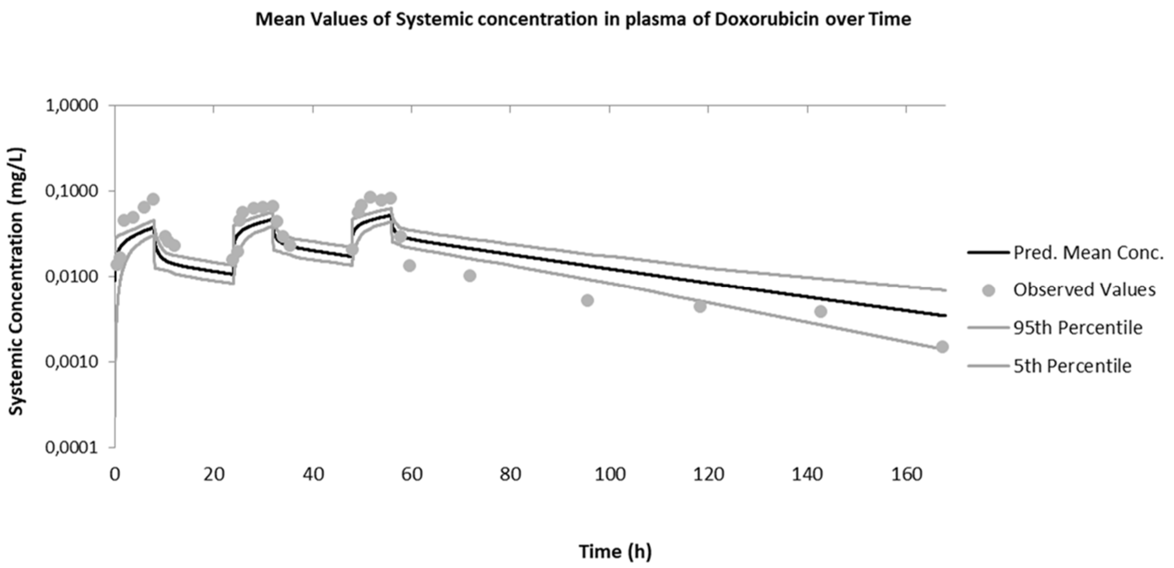

Figure S17. Mean, 95th percentile and 5th percentile of the concentration versus time of DOX for model 3 based on the works of Speth et al. DOX was given as a 3-day IV Infusion over 8 h of 30 mg/m^2 every 24 h.

Model 4

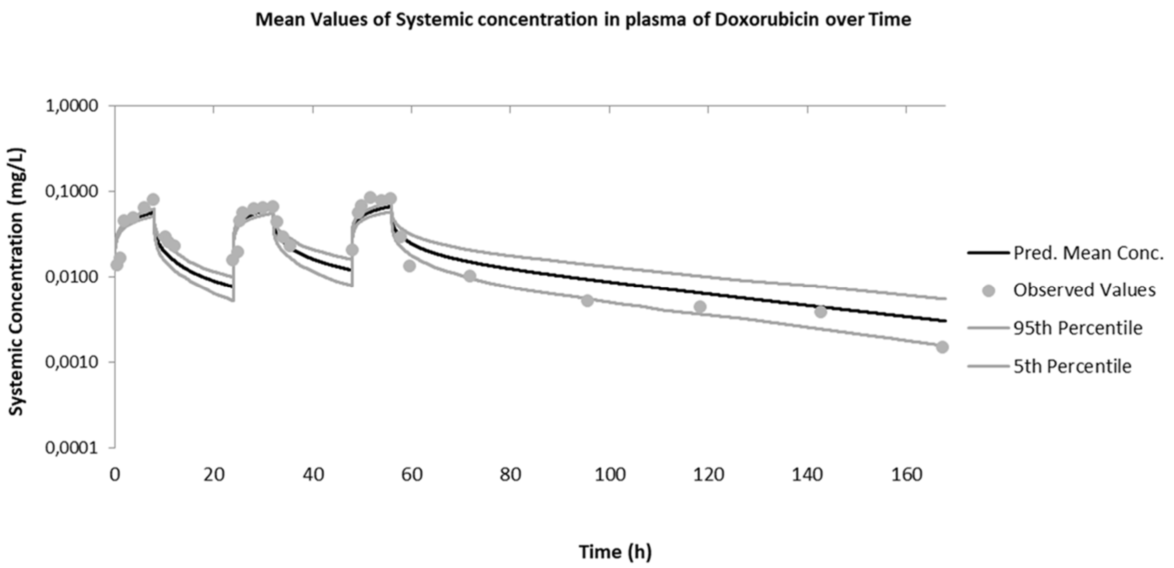

Figure S18. Mean, 95th percentile and 5th percentile of the concentration versus time of DOX for model 4 based on the works of Speth et al. DOX was given as a 3-day IV Infusion over 8 h of 30 mg/m^2 every 24 h.

Model 5

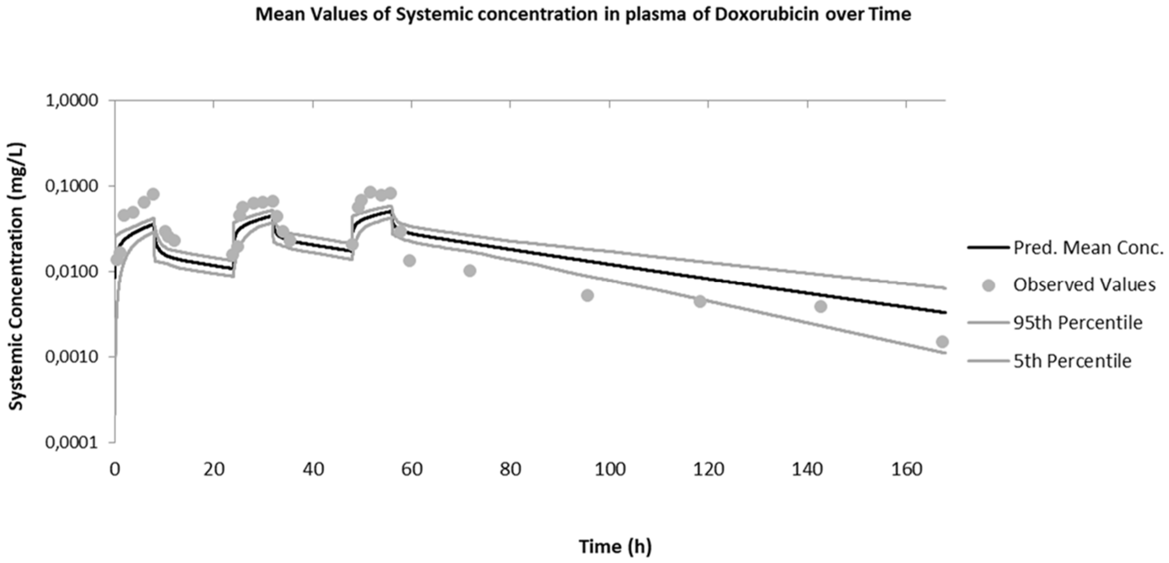

Figure S19. Mean, 95th percentile and 5th percentile of the concentration versus time of DOX for model 5 based on the works of Speth et al. DOX was given as a 3-day IV Infusion over 8 h of 30 mg/m^2 every 24 h.

Model 6

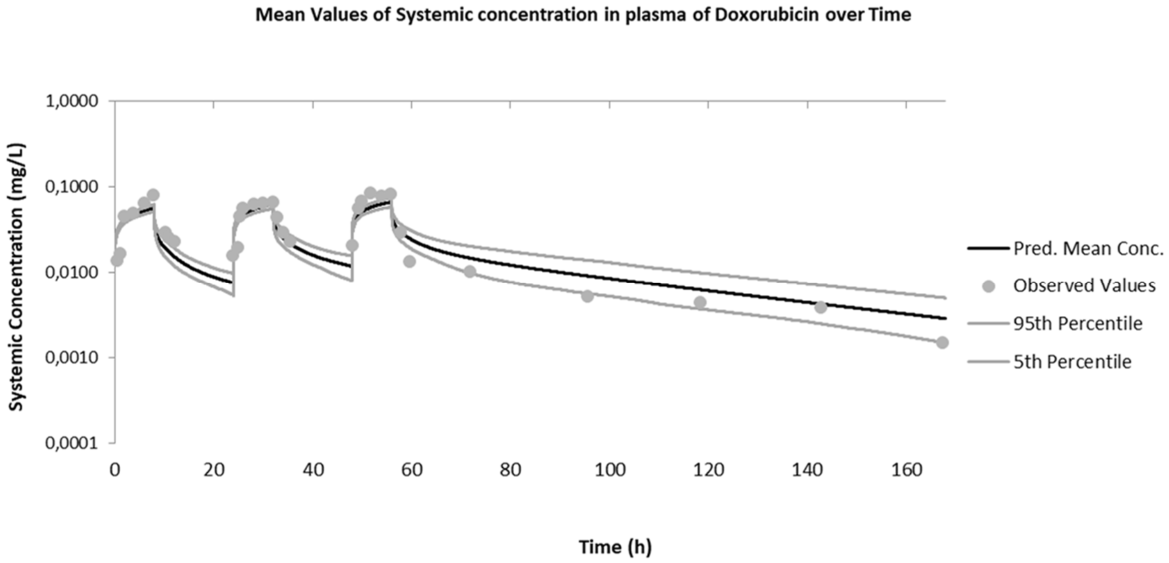

Figure S20. Mean, 95th percentile and 5th percentile of the concentration versus time of DOX for model 6 based on the works of Speth et al. DOX was given as a 3-day IV Infusion over 8 h of 30 mg/m^2 every 24 h.

Model 7

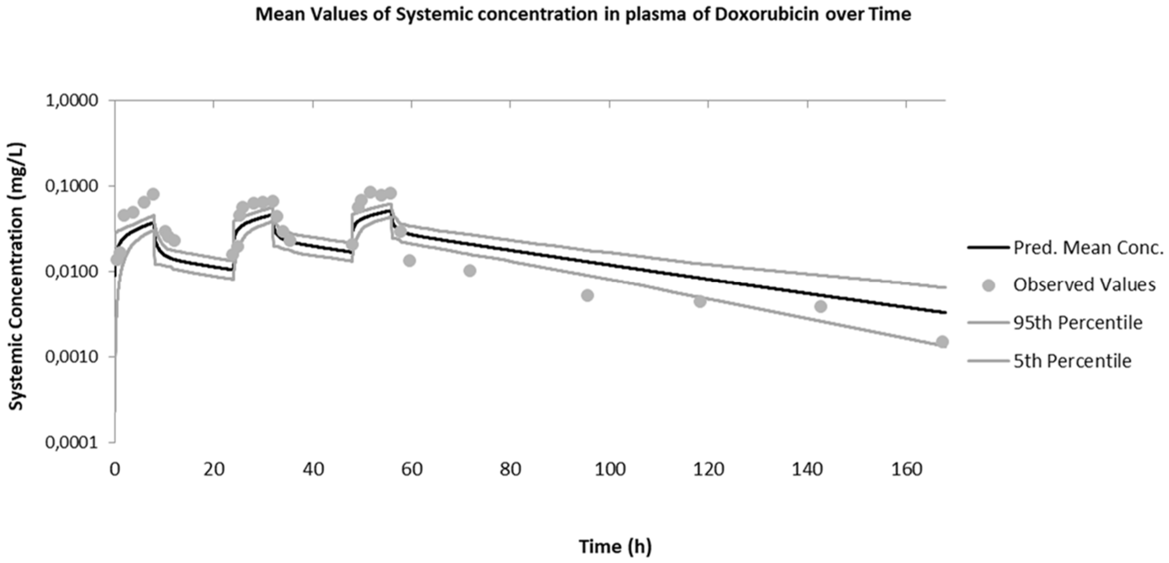

Figure S21. Mean, 95th percentile and 5th percentile of the concentration versus time of DOX for model 7 based on the works of Speth et al. DOX was given as a 3-day IV Infusion over 8 h of 30 mg/m^2 every 24 h.

References

1. Camaggi. C.M. et al. Epirubicin and doxorubicin comparative metabolism and pharmacokinetics. A cross-over study. Cancer Chemother Pharmacol. 1988. **21**(3): p. 221-8.
2. Pawar. S. G. Shevalkar. and P. Vavia. Glucosamine-anchored doxorubicin-loaded targeted nano-niosomes: pharmacokinetic. toxicity and pharmacodynamic evaluation. J Drug Target. 2016. **24**(8): p. 730-43.
3. Johnson. T.N. et al. Changes in liver volume from birth to adulthood: a meta-analysis. Liver Transpl. 2005. **11**(12): p. 1481-93.
4. Heinemann. A. et al.. *Standard liver volume in the Caucasian population.* Liver Transpl Surg. 1999. **5**(5): p. 366-8.
5. Barter. Z.E. et al. Scaling factors for the extrapolation of in vivo metabolic drug clearance from in vitro data: reaching a consensus on values of human microsomal protein and hepatocellularity per gram of liver. Curr Drug Metab. 2007. **8**(1): p. 33-45.
6. Barter. Z.E. et al. Covariation of human microsomal protein per gram of liver with age: absence of influence of operator and sample storage may justify interlaboratory data pooling. Drug Metab Dispos. 2008. **36**(12): p. 2405-9.
